# Reliability of plastid and mitochondrial localisation prediction declines rapidly with the evolutionary distance to the training set increasing

**DOI:** 10.1371/journal.pcbi.1012575

**Published:** 2024-11-11

**Authors:** Sven B. Gould, Jonas Magiera, Carolina García García, Parth K. Raval

**Affiliations:** Institute for Molecular Evolution, Heinrich–Heine–University Düsseldorf, Düsseldorf, Germany; US Army Medical Research and Materiel Command: US Army Medical Research and Development Command, UNITED STATES OF AMERICA

## Abstract

Mitochondria and plastids import thousands of proteins. Their experimental localisation remains a frequent task, but can be resource-intensive and sometimes impossible. Hence, hundreds of studies make use of algorithms that predict a localisation based on a protein’s sequence. Their reliability across evolutionary diverse species is unknown. Here, we evaluate the performance of common algorithms (TargetP, Localizer and WoLFPSORT) for four photosynthetic eukaryotes (*Arabidopsis thaliana*, *Zea mays*, *Physcomitrium patens*, and *Chlamydomonas reinhardtii*) for which experimental plastid and mitochondrial proteome data is available, and 171 eukaryotes using orthology inferences. The match between predictions and experimental data ranges from 75% to as low as 2%. Results worsen as the evolutionary distance between training and query species increases, especially for plant mitochondria for which performance borders on random sampling. Specificity, sensitivity and precision analyses highlight cross-organelle errors and uncover the evolutionary divergence of organelles as the main driver of current performance issues. The results encourage to train the next generation of neural networks on an evolutionary more diverse set of organelle proteins for optimizing performance and reliability.

## Author summary

Recent advancements in genome sequencing and machine learning have been instrumental in solving numerous biological challenges, such as the prediction of the folded state of proteins from sequences alone. An intriguing cell biological challenge is tracking the localization of proteins within cells. For example, some nuclear-encoded proteins localize to mitochondria, while some are sorted to plastids. Experimentally tracking the localization of each protein across thousands of species is laborious. Instead, researchers use machine learning algorithms to predict where proteins are likely to be localized based on their sequence. How reliable are these predictions? We evaluated the reliability of prediction tools across more than a hundred plant species. We found that, as the evolutionary distance between the species used for training the algorithms and those used for testing increases, the accuracy of the predictions declines sharply. This perspective has allowed us to propose new strategies to improve these algorithms. We believe that training more distant plant genome sequences in combination with advances in artificial intelligence–and viewed through an evolutionary lens–will be crucial for developing localization prediction algorithms that are reliable across a wide range of species.

## Introduction

A plant encodes 20–30,000 proteins on average, of which many thousand are targeted to intracellular membrane bound compartments after or during translation [1–3]. The compartments owe their origins to bacterial ancestors directly or indirectly [4–12]. Mitochondria and plastids are of endosymbiotic origin and have transferred a majority of their coding capacity to the nuclear genome in the course of their transition from bacterium to organelle [13–15]. As a consequence, the vast majority of their proteins are translated in the cytosol and need to be imported. Protein translocation-related components of mitochondria such as TOM40, VDAC, TIM22, TIM23-PAM, OXA, SAM, HSP70, or the mitochondrial pre-sequence protease are likely of alphaproteobacterial origin [16–22], while many components of the plastid import machinery such as TOC75, OEP80, TIC20, the TAT pathway and several signal processing peptidases are of cyanobacterial origin [23–34]. Despite their evolutionary independent roots, the import machineries of mitochondria and plastids are united by principles of how they recognize the vast majority of their cargo.

Cytosolically-translated proteins destined for the mitochondrial matrix or the plastid stroma, thousands in sum, carry N-terminal targeting sequences (pNTS for plastid; mNTS for mitochondria) with many similarities and subtle differences. They concern the overall amino acid composition, processing peptidases and translocation motifs, and an overall charge difference among the more N-terminal region, in which mNTSs are enriched in arginine and pNTS are enriched in hydroxylated amino acids [35–39]. The subtle differences are still not fully understood, but determine whether a preprotein is targeted to mitochondria, plastids, or in the case of dual targeted proteins to both compartments simultaneously [40]. Considering the many remaining obstacles of *in vivo* protein localisation (time, resources, overexpression artefacts, impact of the tags on the cargo, or the simple unavailability of transfection methods for non-model systems) [41–47], hundreds of studies rely on algorithms that depend on the difference in NTS features for their localisation prediction. Furthermore, such prediction algorithms are integral parts of widely used databases such as Phytozome [48] or they are nested inside software packages such as InterProScan [49]. Hence, the algorithms are often used routinely, sometimes without a conscious decision to do so, and usually with a lack of knowledge on how reliable they work outside of the species on which they were trained.

*In-silico* localisation predictions from amino acid sequences were implemented concomitant with our understanding of cellular protein sorting [50–54]. Amino acid composition was used to differentiate between intracellular and secreted proteins [55–57], followed by the use of N-terminal features (e.g. charge and hydrophobicity) for signal sequence detection and cleavage site identification [52,58,59]. This channelled into early prediction algorithms such as PSORT [60] that relied on a relatively simple set of ‘if and then’ rules to predict signalling peptides and secreted proteins in Gram-negative bacteria and also included eukaryotes. PSORT II, an early formal expansion [61], incorporated a more sophisticated technique of k-nearest neighbours (kNN), which searches the query against a database of proteins with known localisations and assigns localisation of the nearest neighbours to the query. PSORTb [62,63] introduced machine learning by including support vector machines for accumulating protein sequence features relevant to localisation. This culminated into WOLFPSORT (WPS from here on), one of the first sophisticated machine learning algorithms [64,65]. The algorithm uses approximately 20 features of the query sequence to calculate feature vectors, closest neighbours of which from the database are used for assigning a localization prediction. More than a decade later, the next generation of programs including Localizer and TargetP were released, which profited from more experimental data and advances in supervised machine learning^66,67^. Localizer is a classifier algorithm trained to differentiate between N-terminal regions of known organellar and non-organellar proteins. It abstracts 58 features of proteins from a positive and negative training set and the training process sets a boundary, which is a function of the weighed features. The features from a query are set on a hyperdimensional space and sorted into organelle or non-organelle using the boundary as a reference. TargetP 2.0 is an even more sophisticated algorithm that utilises bidirectional neural networks and multi-attention mechanisms on a network of interconnected, long short-term memory cells [66].

**Fig 1:**
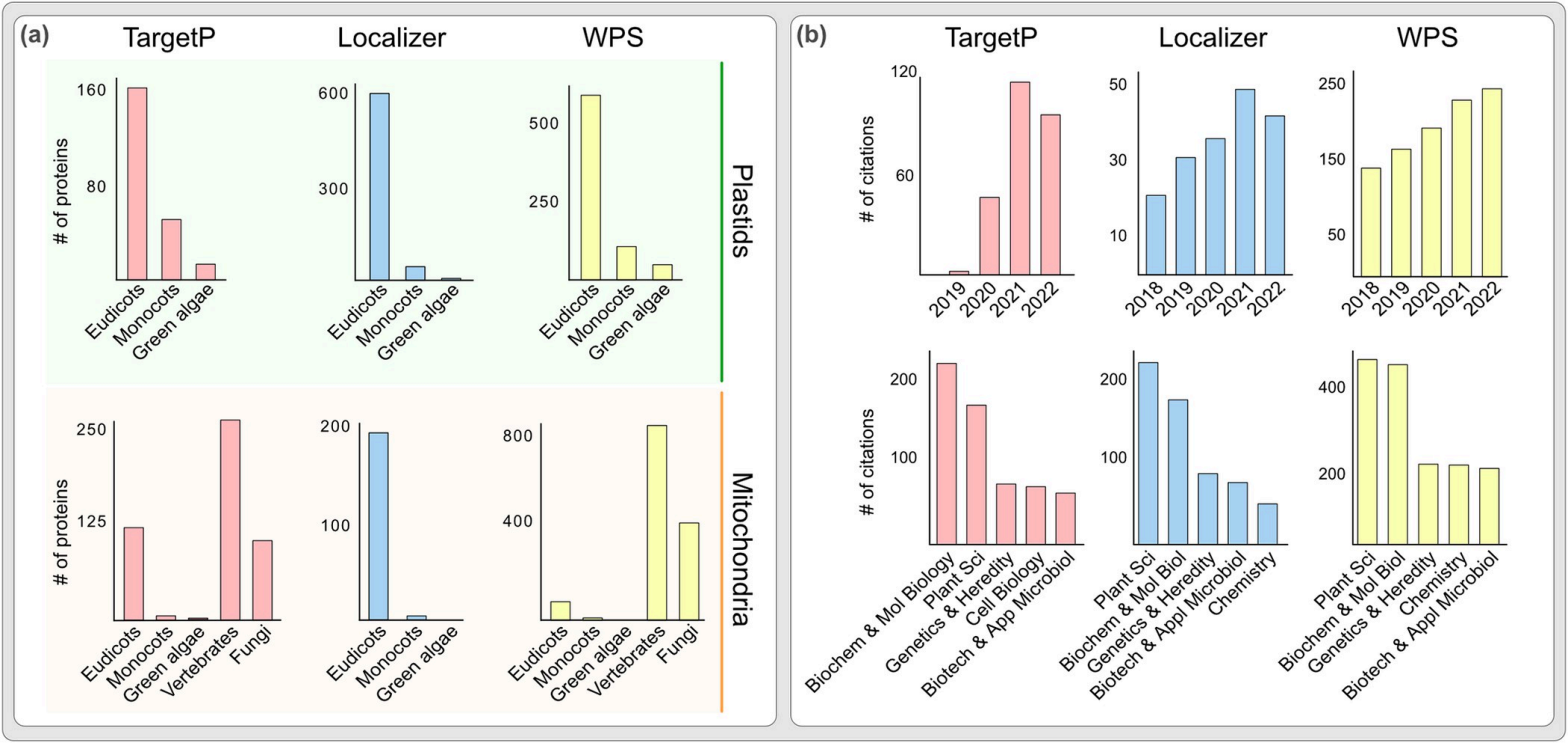
Targeting prediction algorithms are frequently cited across disciplines and rely on a limited training set. **(a)** Taxonomic distribution of plastid and mitochondrial training datasets used for the three commonly used predictions tools TargetP, Localizer and WoLF PSORT (WPS). **(b)** Distribution of citations across different disciplines for the three commonly used predictions tools TargetP, Localizer and WoLF PSORT (WPS) and for a time period ranging from 2018 until 2022. Numbers according to the Web of Science.

Apart from the training and sorting operations, the training datasets themselves also vary (Fig 1A). WPS for example used a database of 2004 (Uniprot v45.0), a time at which no genomes for bryophytes, ferns, let alone streptophyte algae or multiple organelle proteomes were available. Its training dataset was almost exclusively based on eudicot (for plastid) and animal (for mitochondria) sequences and the proteins were selected based on their annotation from the gene ontology database (GO; evidence codes: TAS, IDA, IMP; cut-off 12.4.2004). Two of these evidence codes (TAS and IMP) are indirect [67] and when used as a starting point, prone to multiplying errors. Localizer was trained on several hundred Viridiplantae organelle proteins from Uniprot (database until March 2016) and validated on the cropPal dataset (barley, wheat, rice, maize) as well as Uniprot Viridiplantae organelle proteins that were added between March and September of 2016. Of these Viridiplantae proteins, a vast majority was of eudicot origin. Tools such as cropPAL or SUBAcon (SUBcellular localisation database for Arabidopsis Consensus) significantly increase localisation prediction reliability, but only for selected eudicots on which they were optimized [43,68–70]. TargetP uses a relatively recent training data, including some green algal proteins, but again leaning heavily towards eudicots.

While vastly different in underlying algorithms and species data, TargetP, Localizer and WolFPSORT are among the algorithms with a superior reported accuracy. They are used abundantly across disciplines (Fig 1B), but are rarely benchmarked systematically across a wide range of species. Therefore, the impact of the skewed training on the performance and reliability of these algorithms outside angiosperms are unexplored. We made use of available, experimentally verified plant proteomes of mitochondria and plastids as well as protein clustering to investigate the reliability of these algorithms across species ranging from algae, across bryophytes and to angiosperms and organism with increasing research interest [71–77]. Our analysis brings forth deficiencies of these algorithms, caused by a combination of their inherent *modus operandi*, a lack of training on a diverse dataset, and the evolutionary dynamic nature of plant organelles [78]. Tracing the error sources allows to sketch an approach towards developing better algorithms that are capable of serving the diversity of the plant kingdom.

## Results

### Algorithms perform poorly outside of their training species

**Fig 2:**
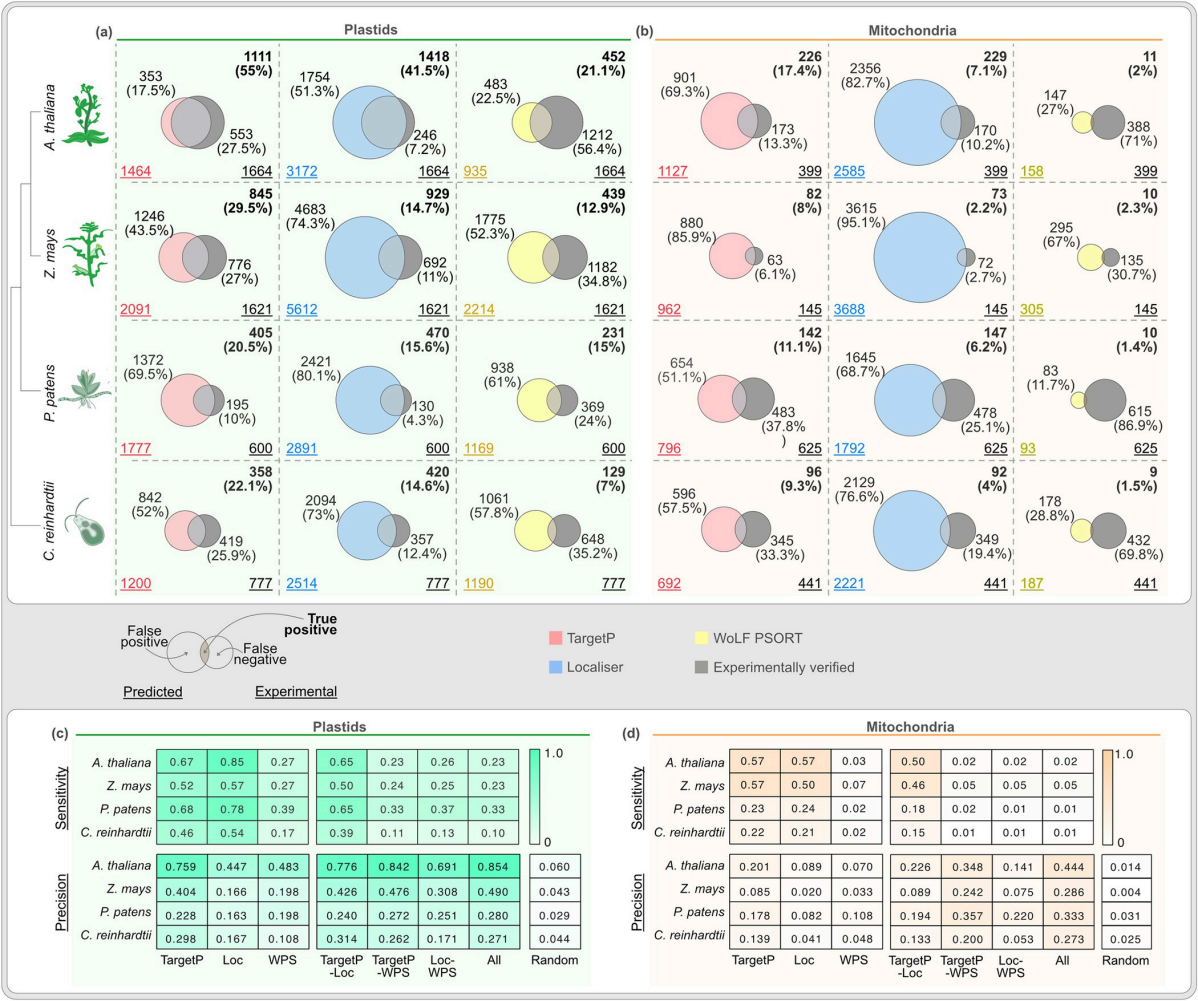
Performance of algorithms outside the training species. Comparison of predicted versus experimentally localised plastid **(a)** and mitochondrial **(b)** proteome numbers. Each Venn diagram of the top panel shows an overlap between predicted (left circles, colour-coded based on the algorithms used) and experimentally verified organelle proteomes (right circles, grey). The underscored numbers in the bottom corners show the total number of predicted (bottom left) and experimentally confirmed proteins (bottom right). The numbers of proteins that overlap (true positives) are provided in the top right corner in bold, while the numbers of non-overlapping false positives and negatives are shown next to each circle. See also the key for the Venn diagrams on the bottom left. Sensitivity, specificity and precision of individual algorithms and their combinations for plastid **(c)** and mitochondria **(d)**.

First, we compared the organelle proteomes predicted by the algorithms (the *in-silico* proteomes) with those of experimentally verified organelle proteomes (the *in-vivo* proteomes). Across species, *in-silico* proteomes comprise 3–15% of the proteins encoded by the genome of a given species, in contrast to the *in-vivo* numbers that usually range from 5–10% (S1 Fig). Overlaps between *in-silico* and *in-vivo* proteomes show a substantial false positive rate except for the *in-silico* plastid proteome predicted for *Arabidopsis* by TargetP (Fig 2A and 2B). Localizer and WPS show larger fractions of false positives than TargetP, especially for mitochondria (Fig 2B). The smallest overlap between *in-silico* and *in-vivo* proteomes are found for WPS. False negatives are generally predicted fewer on average than false positives, but still to a substantial number (Fig 2A and 2B). The sensitivity of TargetP and Localizer are similar, above 0.5 for plastid (i.e., correctly identifying more than half of the plastid proteins) and below 0.5 for mitochondria, whereas that of WPS is 0.3 or lower (Fig 2C and 2D). Since 2–5% of the proteins encoded in a nuclear genome have been localised to mitochondria or plastids *in-vivo* through proteomics or tagging (S1 Fig), a random sampling has a precision of 0.02–0.05; a perfect algorithm should have a precision of or close to 1. Between these two theoretical extremes, established algorithms currently perform closer to random sampling than to the best-case scenario, especially for mitochondria. The best improvement over a random prediction is observed for TargetP on *Arabidopsis* data, which however shifts ever closer to random the greater the evolutionary distance from *Arabidopsis* gets.

Combinations of algorithms reflect similar trends, where TargetP and Localizer together perform marginally better than each individually, as previously reported [79], albeit confined to the angiosperm plastid (Fig 2C). For mitochondria, the same combination captured less than 50% of verified proteins across species and any other combination captured less than 5% due the poor performance of WPS (Fig 2D). The precision was high in *Arabidopsis* for all combinations, too, but declined moving towards *Chlamydomonas* and regardless of combination (Fig 2C and 2D). To summarize, the predictions (for any individual algorithm or any combination) are more reliable for angiosperms and with a rapidly declining reliability with respect to algae and bryophytes (Fig 2).

### Prediction performance declines as a function of evolutionary distance from the training data

**Fig 3:**
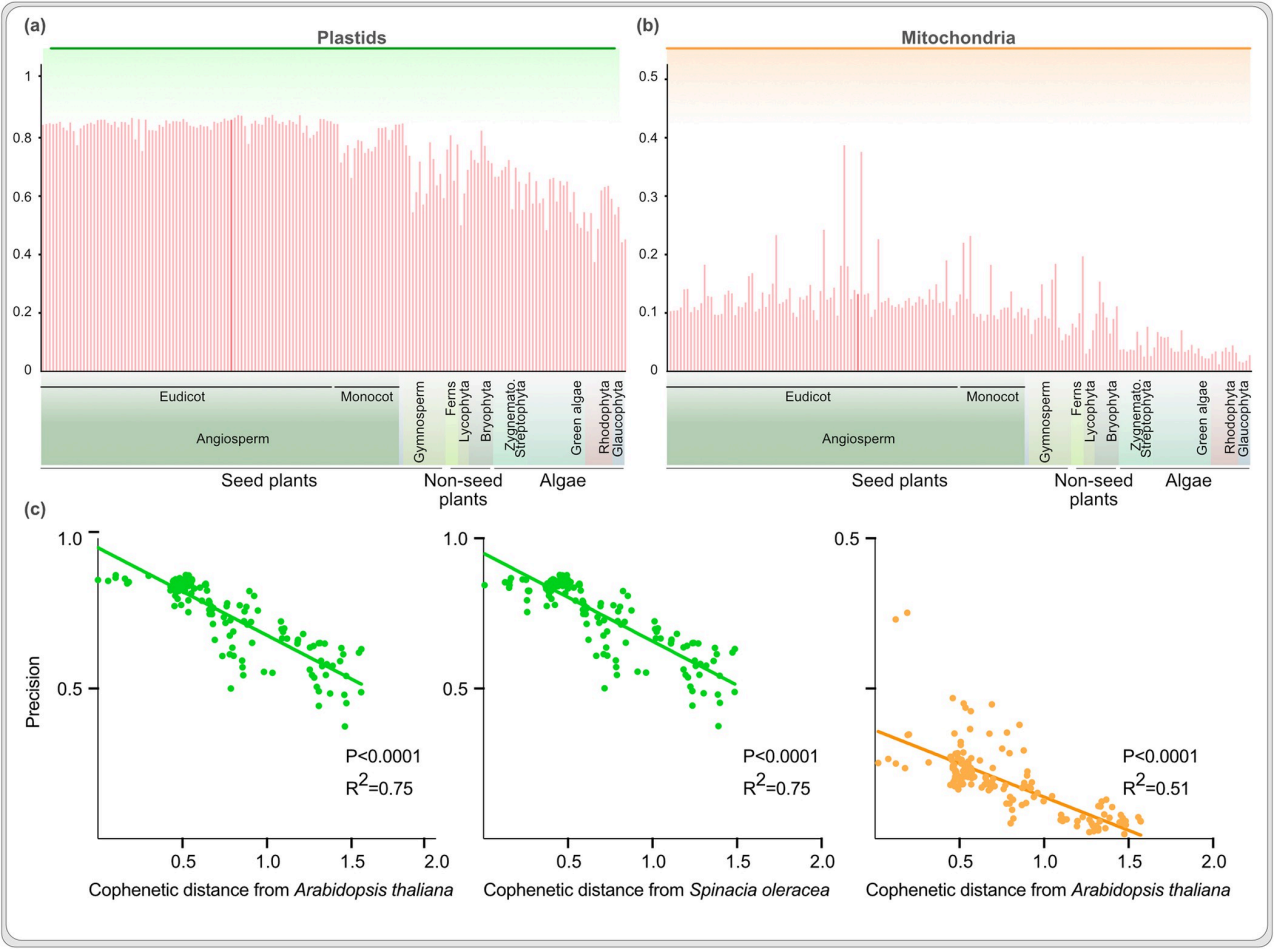
Strong negative correlation between the precision of algorithms and the evolutionary distance from the training data. Precision of TargetP across eukaryotes for plastid **(a)** and mitochondria **(b)**; *A*. *thaliana* is shown in darker shade. Taxonomic classification of test species is shown on the X axis, skewed towards eudicots due to genome sequence availability but similar to the training data (Figs 1A and S5). (c) Precision of TargetP as a function of evolutionary distance between the training species and 171 test genomes (plastid in green and mitochondria in orange).

To expand benchmarking across a larger evolutionary scale, we used TargetP (the best performing among the three) to predict organelle proteomes for 171 photosynthetic eukaryotes, for which genomes are available. Since organelle proteomes are scarce, we utilised orthology inferred organelle proteomes based on their sequence similarity with experimentally validated organelle proteomes [78]. Around 70–80% of predicted plastid proteins in eudicots could be validated by orthology based predictions (Fig 3 and S2 Table). Precision is lower for eudicot land plant sister lineages and algae, however, in accordance with patterns observed across the four species for which there is proteome data available (Fig 2). The precision for mitochondrial protein is lower, including for *Arabidopsis* (and related eudicots) and below 10% for algae. This large and diverse sample size allowed us to systematically and quantitatively test the impact of evolutionary distance between training and test species on the performance of the algorithms. TargetP is trained on 227 plastid and 499 mitochondrial proteins almost exclusively of eudicot or metazoan origin (S5 Fig). We calculated cophenetic (evolutionary) distances for each of the 171 test species from the most prominent training species, which reveals a significant negative corelation between the precision of algorithms across test species and the evolutionary distance of the test species from the training species. It underscores the need for an algorithm, whose performance is optimized with respect to evolutionary diversity. To this end, we next investigated sources of the prediction errors.

### The training bias of algorithms causes *in-silico* cross-organelle contamination

**Fig 4:**
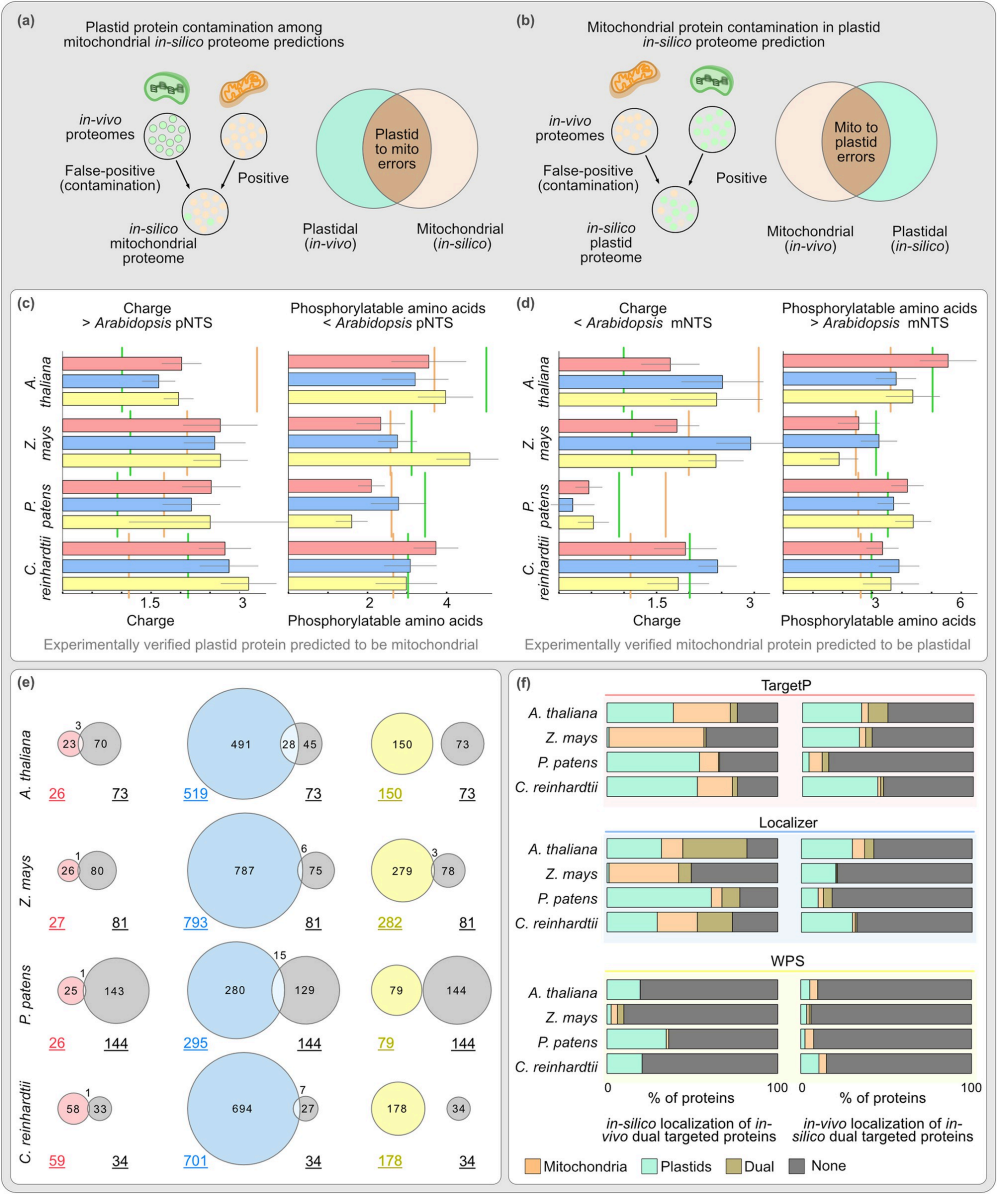
Cross-organelle errors in proteome prediction due to physio-chemical properties of the NTS. Cross organelle prediction errors could be either because an *in-vivo* plastid protein is *in-silico* mitochondria localised **(a)** or *vice versa* **(b)**. The overlaps between cross-organelle *in-vivo* and *in-silico* proteomes identifies these predictions errors. Analysis of the first 20 amino acids of pNTSs incorrectly predicted to be mitochondrial **(c)** and *vice versa* **(d)**. Average charge and phosphorylatable amino acids for NTS from all verified organelle proteins of each species are indicated by vertical green (pNTS) and orange (mNTS) lines. Error bars indicate standard error of mean (N = 4–331, S2 Fig). **(e)** Overlap between predicted (left) and experimentally localised (right, in grey) dual targeted proteins. **(f)** Predicted (*in-silico)* intracellular localisation of experimentally verified (*in-vivo*) dual targeted proteins (left column) and experimentally verified (*in-vivo*) intracellular localisation of proteins that are predicted (*in-silico*) to be dual targeted (right column).

One likely source of false positives is the errors between the two organelles, caused also by the similarities in how their protein import machineries evolved. For example, a plastid protein can contaminate an *in-silico* mitochondrial proteome (Fig 4A) or vice versa (Fig 4B). Such errors can be quantified by overlapping the *in-vivo* proteome of one organelle with the *in-silico* proteome of the another: an overlap between the *in-vivo* plastid proteome and the *in-silico* mitochondrial proteome, highlights those plastid proteins that “contaminated” the *in-silico* mitochondrial proteome (Fig 4A). We observed that on average about a hundred or more plastid proteins were found across the four species in the *in-silico* mitochondrial proteomes (more frequently so with Localizer, in particular for the bryophyte and alga, S2 Fig) and a smaller number of mitochondrial proteins were identified in the *in-silico* plastid proteomes.

While NTSs of plastid and mitochondrial proteins share similarities, an mNTS contains a statistically significant higher net positive charge, while pNTSs contain a high number of serine and threonine residues among their first 20 amino acids [36]. It seems these differences became more pronounced later in plant evolution, since they are most striking in the angiosperms (Fig 4C and 4D, vertical green and orange lines). This is a good time to remember that more than 95% of discussed training datasets come from angiosperms (Fig 1A). Algorithms are inclined to sort NTSs based on these features and any NTS that deviates would be prone to an erroneous cross-organelle prediction, declining the performance of the algorithm. Indeed, NTSs of plastid proteins that showed a higher charge and/or a lower number of phosphorylatable amino acids than the average *Arabidopsis* pNTS, were predicted to be mitochondrial (Fig 4C) and NTSs of mitochondrial proteins that showed a lower charge and/or higher number of phosphorylatable amino acids than the average *Arabidopsis* mNTS were predicted to be plastid proteins (Fig 4D). These differences underscore that algorithms are trained to recognise and sort evolutionary late angiosperm targeting sequences, a bias that increases the error rate when facing proteins of algae and early branching plant species such as bryophytes.

The substantial number of cross-organelle prediction errors motivated us to investigate the predictability of proteins that are *in vivo* targeted to both, plastid and mitochondria. More than hundred such dually targeted proteins are identified in *Arabidopsis* [40], the plant proteomes of plastids and mitochondria corroborate such numbers and that is how we treated all proteins that overlapped in the proteome analyses. Algorithms can also predict the same protein to be plastid and mitochondria localised, either explicitly (by listing both these compartments) or implicitly (by providing similar probability scores for these two compartments). We considered such cases as predicted dual targeted proteins. *In-vivo* and *in-silico* dual targeted proteins hardly overlap, with hundreds of false positive and false negatives (Fig 4E). Except for maize, TargetP predicted most of the experimentally dual localized proteins (i.e. plastid and mitochondrion) to be only plastid localized or not to be organellar at all (Fig 4E and 4F). Localizer performed better than the other two with respect to quantity, but at the substantial cost of hundreds of false positives, and WPS failed to predict dual targeted proteins altogether. On the whole, all algorithms perform poorly on this task, sorting experimentally dual targeted proteins to only the plastid or no organelle at all, while also labelling non-organellar or plastid proteins falsely as being dual targeted likely as a result of cross-organelle errors (Figs 4A–4D and S2).

In summary, a combination of training bias and the evolution of targeting sequences ever since the origin of eukaryotes with mitochondria culminates into cross-organelle errors, which also affect the predictability of the dual targeted proteins.

### Evolutionary dynamics and the diversity of organelles contribute to prediction inaccuracy

The endosymbiotic organelles of algae and plants have been co-evolving for over a billion-years and their proteomes continue to change and adapt [78,80,81]. During plant terrestrialization for instance, the plastid proteome of the algal ancestor expanded from a few hundred to that of the angiosperm plastid housing about 1500 proteins [78]. The algorithms predict there to be 1000 to 2000 plastid (and mitochondrial) organellar proteins even outside of angiosperms, 25% or less of which appear to be true positives (Fig 2). Together with the general pattern of the prediction performance worsening with the evolutionary distance to model angiosperms increasing, it prompted us to consider evolutionary dynamics of organelle proteomes as another error source.

**Fig 5:**
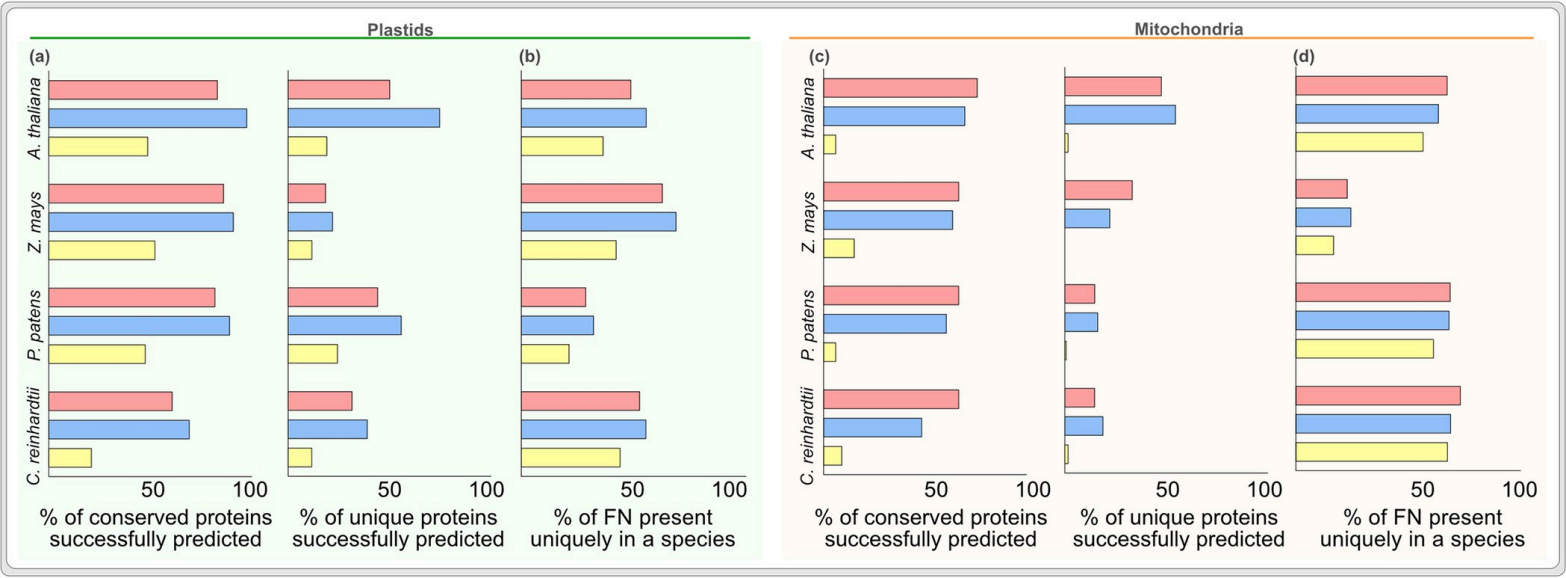
Success rate of predicting unique versus conserved organelle proteins. Success rate (sensitivity) of predicting experimentally verified conserved and unique proteins for **(a)** plastids and **(c)** mitochondria. All proteins from each species were sorted into conserved or unique based on sequence-based protein clustering (see methods, S3 Fig). Of the total plastid and mitochondrial false negatives (from Fig 2A and 2B), the number of proteins that were unique to a given species are shown for plastids (b) and mitochondria (d).

We clustered all proteins from the four species into protein families [82], filtered the experimentally verified organelle protein families, and sorted them to be conserved (present in all four species) or to be unique (present in only one species) (S3 Fig and S1 Table). Around 150 protein families were found to be conserved across all proteomes, whereas a few hundred were unique. TargetP and Localizer missed around 30% of the conserved proteins, and WPS missed more (Fig 5A). For the unique plastid proteins, TargetP and Localizer performed well for *Arabidopsis* with declining success for the other species. WPS missed more than 75% of the unique proteins across the species (Fig 5A). For the conserved mitochondrial protein families, Localizer and TargetP predicted 50–70% correctly, whereas WPS missed more than 90% (Fig 5C). For mitochondria-unique proteins, the success rate ranged from 20–50% for Localizer and TargetP in *Arabidopsis* and other species, while WPS missed more than 90% across the species (Fig 5C). More than half of all protein missed out across the algorithms (i.e. false negatives of Fig 2), were present in only one of a given species (Fig 5B and 5d) and likely missed because of a lack of diverse training datasets. With the growing notion of organelle ‘pan-proteomes’, i.e. organelle proteins present in selected species or organelle sub-types [78,79,81,83–87], our analysis shows that algorithms are inadequate at capturing this pan-proteome nature or even the distant homologues of conserved proteins. To cover the species-specific organelle proteins and possibly the pan-proteome, future algorithms could be trained on missed proteins from across (Fig 2A and 2B) and from within species [88]. Algorithms could then sort predictions into a core- and pan-proteome, assigning credence and error margins likewise.

## Discussion

After its cytosolic translation, a plant protein needs to be targeted to the correct compartment if it is not to remain in the cytosol. Machine learning algorithms are used abundantly to determine where proteins are targeted, but they are trained on phylogenetically constrictive datasets (Fig 1A). They are often benchmarked on a limited number of species and, like the study at hand, identify TargetP among the most reliable prediction tools [68,69,79]. We benchmarked over a hundred photosynthetic eukaryotes including algae, using proteomes from four diverse representative species. The three widely used algorithms evaluated here perform poorly outside of model angiosperms, especially for mitochondrial cargo, for which the targeting prediction is only slightly better than random sampling. TargetP, the best performing among the three, has a fifty-fifty chance of sorting an algal plastid protein correctly and twice the chance of predicting a false positive. For mitochondria, the error margins are worse. For WPS, the most cited of the three analysed (Fig 1B), the chances of a wrong prediction are several times higher for plastid- and tens of times higher for mitochondrial proteins.

**Fig 6:**
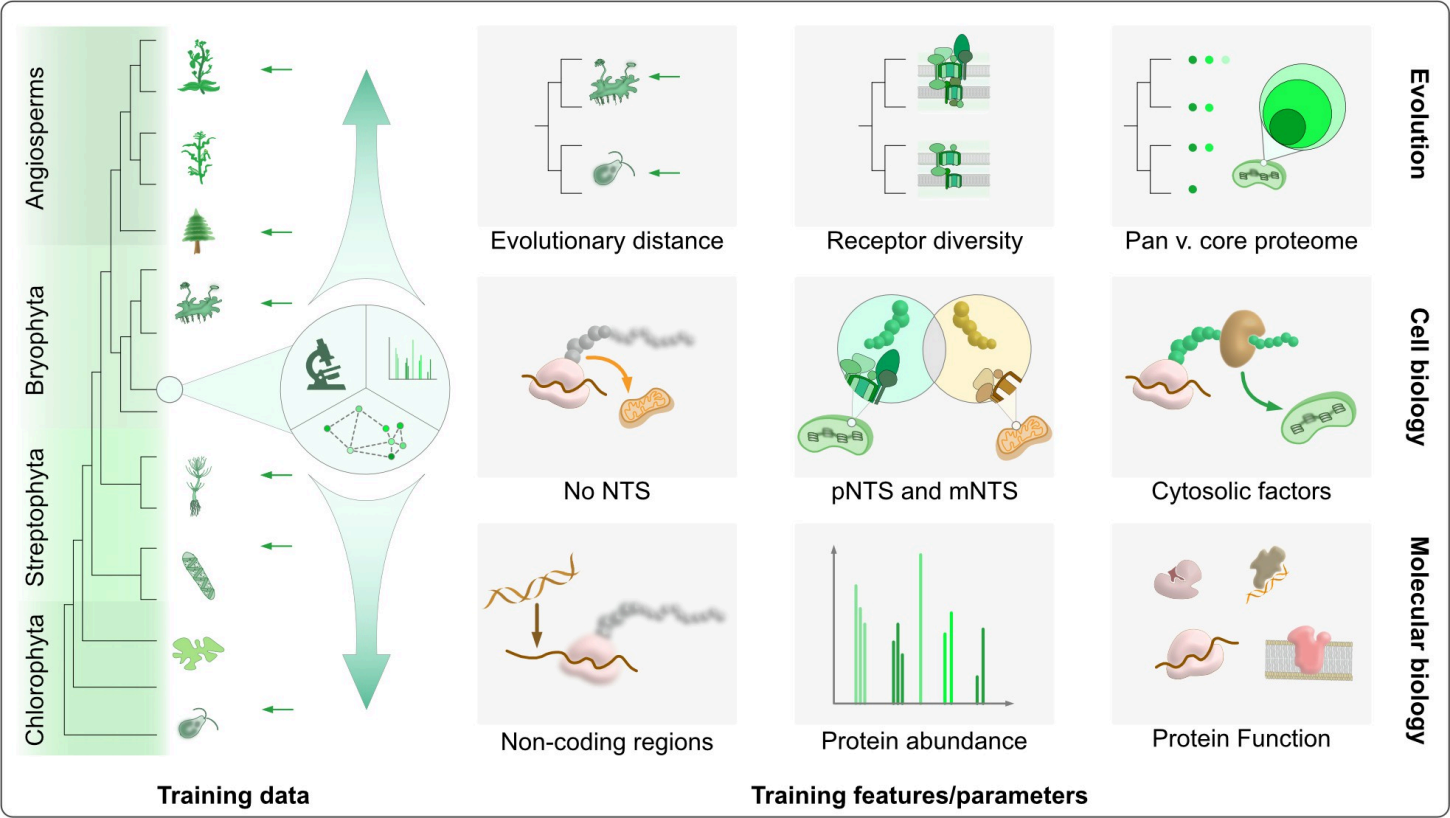
A framework for improving localisation prediction algorithms. Strategies to improve prediction reliability involve changes in the curation of the training data as well as the training procedure. The training data should ideally be collected from a range of diverse species and for each be based on different experimental techniques that support a training protein’s localisation (e.g. reporters, mass spectrometry, coexpression, interactions). Proteins with non-canonical internal motifs, or those dually targeted need to be taken into account (as they help to better distinguish between pNTS and mNTS features) and validated data could be sorted according to whether it is part of a core- or pan-proteome. Classifiers on which the algorithms are trained could include parameters such as the evolutionary distance of a species, non-coding regions, or a protein’s abundance as a currently neglected factor. One can expect that the combination of multi-dimensional parameters from evolutionary biology, cell biology and molecular biology on evolutionary diverse species will significantly improve the next generation of machine leaning algorithms that serve localisation (and function) predictions.

Such systematic error margins are a real issue, yet the output is trusted across individual studies directly (Fig 1B) or indirectly through being part of software packages and databases that cover genomes from hundreds of diverse species. Their genome annotations, however, receive localisation predictions from the same set of algorithms, but up to 70–80% of them can be wrong (Fig 3). While some of these ‘false positives’ can be attributed to experimental errors–the algorithms outperforming the experiments–this fraction is likely small. To reconcile experimental errors and contradictions [45–47], a combination of multiple experimental approaches under comprehensive projects such as SUBA or cropPAL [43,68–70,89] would be a first step to curate training data for algorithms incorporating chloro-, strepto- and bryophyte species (Fig 6). Algorithms thus trained on phylogenetically diverse datasets would improve reliability of large datasets, while being equally useful to diverse areas of fundamental and applied life sciences (Fig 1B).

Evaluating the error source in light of the cellular complexity and evolutionary cell biology of plants, allows to sketch improvement strategies for future algorithms. More than a billion years of co-evolution has resulted in plastid and mitochondrial proteomes and their import machinery, nuances of which affect the predictability of protein sorting. For instance, likely due to a selection pressure against plastid mistargeting, mitochondrial protein import evolved specific receptors such as TOM20 and TOM70 [90–94] that are unique to plant mitochondria. They have binding sites for cargo that differs from that of animal mitochondria [95–97]. Moreover, some of the predominant mNTS features from yeast (e.g. β-sheets) [98] are extremely rare in plant mNTSs and are rather similar to critical features of pNTS [99]. Therefore, including yeast mNTS in the training data sets generates false prediction to plastid in plants [100]. Such details are often not accounted for by the algorithms that are hitherto trained almost exclusively on animal and yeast sequences (Fig 1A). Consequently, algorithms require an upgrade to be able to predict plant mitochondrial proteomes and training them on plant mitochondrial proteins, and accounting for the receptor platform differences, is essential (Fig 6).

The impact of organelle co-evolution appears to be more pronounced in angiosperms sequences (the training dataset), which evolved features different from other clades, such as longer pNTSs and different physicochemical properties of NTSs in general [35,101–103]. The details of NTSs are mostly studied in a few angiosperms [104–108], however, and in league with the skewed training (Fig 1A) compromises the performance of algorithms outside of angiosperms. For instance, we utilised BaCeLlo [109], trained on *M*. *musculus*, *S*. *cerevisiae*, *C*. *elegance*, and *A*. *thaliana*, on false negatives (i.e. organelle proteins missed by each of the three algorithm) from *Physcomitrium*. It sorted up to 50% of them to mitochondria, regardless of their experimental localisation (S6 Fig). This further underscores that an algorithm trained on more than one species can also perform poorly outside of angiosperms, when the training focusses on animal and angiosperm sequences alone. A better understanding of NTSs outside of angiosperms remains a bottleneck for developing better algorithms, as much as it remains an unchartered territory in the field of protein import evolution.

Some NTSs are ambiguous and identified equally well by the import machineries of mitochondria and plastids. Although these dual targeted proteins are small in number, they play a key role in information processing [110,111] and have been theorized to reroute whole metabolic pathways [112]. The process of dual targeting appears to be conserved [113,114], rarely lost [113] and can arise by small changes in the NTS [115]. Therefore, it is likely to be common across species, but outside of the model systems the identification of dually targeted proteins is limited. Algorithms are currently of little use in this respect, as they assign dual targeted proteins usually only to the plastid, sort plastid proteins to mitochondria as reported previously [116], or falsely predict many sole plastid proteins to be dually localised. *In vitro* protein import assays with purified organelles also localise many plastid proteins to plastid and mitochondria both, which complicates the matter [117–120]. Ambiguous Targeting Predictor (ATP), an early algorithm tailored towards dual targeting [121], predicted ca. 500 *Arabidopsis* proteins to be dual targeted, of which only 30 have been experimentally verified to date (S7 Fig). CropPAL [68] predicts several hundreds to a few thousand dually targeted proteins for six species, of which <5% are experimentally supported by the same database (S8 Fig). In a previous study, SUBAcon predicted around 30 proteins to localise to both mitochondria and plastids [69], a performance comparable to that of TargetP (Fig 2A). This further underscores that algorithms either under- or massively overpredict dual targeted proteins and this remains a crucial challenge. Such *in vitro* and *in silico* errors limit our understanding of in vivo dual targeting mechanisms. Studying protein dual targeting outside of the established model systems would elucidate general strategies of dual targeting. In the interim, explicitly training algorithms on verified dual targeted proteins could help to identify targets for experimental investigation.

Our analysis also shows that prediction reliability at large declines significantly, when phylogenetically diverse species come into play. In contrast to previous benchmarks, we systematically quantify the extent of error, as test data diverge from training data using phylogenetic distance. Such quantifications allow algorithms to provide a confidence interval–a feature largely missing–based on the evolutionary distance between the training and test data. They can also be used to reject a query, if the evolutionary distance value crosses a certain threshold. The next step could be to systematically use evolutionary distance as a parameter in machine learning, weight of which can be assigned during training-testing iterations on mitochondrial and plastid proteomes from diverse species. When doing so, and in the absence of proteome data, one could commence with canonical and universally accepted organellar marker proteins. Lastly, most algorithms assume the presence of an NTS and attempt to sort a query to organelles. N-terminal targeting peptide-independent import, however, is known and the nature of cargo recognition often more involved [122]. This presents another source of error and requires to predict a localisation on classifiers independent of targeting sequence features alone and they could include e.g. homology, GO or KEGG annotations, or even promoter length [123].

Apart from prediction errors, organelle proteomes can vary also across closely related sister species. For example, a systematic comparison of organelle proteomes between several eudicots and monocots showed that proteomes within crops were more similar to each other than to *Arabidopsis*, highlighting the clade-specific nature of organelle proteomes [70]. This study also highlighted that functions influence how conserved the localisation is across species (and that e.g. the localisation of proteins involved in metabolism are less conserved). Such examples motivates algorithms tailored to a given species [123,124] or clade [116,125], but recent computational power and AI advances encourage us to try the opposite and attempt to develop more generalised algorithms, which abstract clade-specific peculiarities. Moreover, not all proteins are equally abundant in organelles, but they often contribute equally to the training process of algorithms. It is conceivable that NTSs have evolved differences based on protein abundance. Inclusion of relative abundance of proteins in the training process might improve the predictions and reveal novel strategies of protein sorting. Applying such strategies to train algorithms on a diverse set of species (Fig 6) would increase their generalisability.

In conclusion, as advances in in proteomics [126,127], genomics [75,77,128–132], and machine learning [133,134] set a stage for future prediction algorithms, our analysis serves as a reminder that considering evolutionary diversity is key, also to a better modelling of protein sorting.

## Methods

### Algorithms

All algorithms were installed on a local server supported by the ZIM at the HHU Düsseldorf. Full proteomes were analyzed using TargetP 2.0 (https://services.healthtech.dtu.dk/services/TargetP-2.0/) with the setting ‘pl’ (plant derived); with Localizer 1.0.4 (https://localizer.csiro.au/software.html) with Python 2.7 and setting ‘-p’; WPS 0.2 (https://github.com/fmaguire/WoLFPSort) with setting ‘plant’. The outputs were processed using the script ‘Algorithm_predicted_proteins.py’ and ‘batch_process_targetP.py’ (for targetP across eukaryotes in Fig 3). The dual targeted proteins were retrieved using the script ‘dual_targeting_prediction.py’. The number of citations for each algorithm were retrieved from the Web of Science.

### Source genomes and organelle proteomes

Genomes of all chloroplastida species were downloaded from Kyoto Encyclopedia of Genes and Genomes (KEGG) [135]. Experimental organelle proteomes were retrieved from published literature and database as follows: *Chlamydomonas reinhardtii* (chlorophyte algae) [136,137], *Physcomitrium patens* (bryophyte) [138], *Zea mays* (monocot) [42], *Arabidopsis thaliana* (eudicot) [42]

### Evaluation of algorithms

We evaluated the performance in species from four diverse chloroplastida species. A protein present in verified proteome and absent in prediction was categorised as false negative. A protein absent in verified proteome and present in prediction was categorised as false positive. A protein present in both, verified and experimental, proteome was categorised as true positive. Sensitivity (i.e. true positive rate) was calculated as a ratio of true positive and true positive + false negative.


$$Sensitivity=\frac{TP}{TP+FN}$$

Precision was calculated as a ratio of true positive and all predictions.


$$Precision=\frac{TP}{TP+FP}$$

The script ‘Sensitivity_precision.py’ was used for the calculation of sensitivity and precision and the Venn diagrams were generated using ‘Venn_diagrams.py’.

For a combinatorial approach, organelle proteomes were predicted individual by each algorithm and proteins present in the prediction of both or all three algorithms were filtered for further evaluation against experimental proteome. TargetP2.0 predicted ‘thylakoid’ proteins as a category distinct from ‘chloroplast’ and therefore around 100 thylakoid proteins were not included under ‘chloroplast predicted’ category. Inclusion of these proteins do not change broad patterns by more than a few percentage (S4 Fig, as compared to Fig 1A).

### Protein family clustering and annotation

Whole proteomes of all species were clustered into protein families using Orthofinder version 2.5.4 [82]. Source genomes of all species was taken from KEGG [135].

### Analysis of N-terminal targeting sequences and prediction of the dual targeted proteins

The first 20 amino acids of each protein were retrieved from the whole genome assemblies using the script ‘get_first_20AA.py’. Charge was determined by assigning -1 to D,E; +1 to K,R; +0.5 to H and 0 to the rest of the amino acids. The total number of serine and threonine were counted as phosphorylatable amino acids. Both these features were retrieved using the script ‘charge_phospho.py’. The verified dual targeted proteins were inferred from overlapping the experimental proteomes of mitochondria and plastid for each species. TargetP sorts proteins to only one intracellular localisation, which gets the highest probability. However, if probability of mitochondria and plastid both were above 0.35, we considered that protein to be dually targeted. WPS and Localizer predicted more than one localization explicitly, and hence proteins predicted as plastid and mitochondria, were labelled dually targeted. This was done using the script ‘dual_targeting_prediction.py’. The experimental localisation of predicted dual targeted proteins and the predicted localisation of experimentally dual targeted proteins were received by the scripts ‘Exp_dual_insilico_Loc.py’ and ‘Exp_loc_of_predicted_DTP.py’.

### Corelation between evolutionary distance between training and test species and precision

The genomes of 202 eukaryotes and its phylogeny, along with inferred plastid and mitochondria localisation protein families were retrieved from previous study [78]. TargetP analysis and calculation of prevision was performed as described above. The protein identification numbers of the training data and their species details were retrieved from TaregtP 2.0 (https://services.healthtech.dtu.dk/services/TargetP-2.0/) and uniprot (http://uniprot.org/) websites respectively. The frequency at which different species were represented in the training data was calculated and top two plant species were chosen for evolutionary distance analysis. Their phylogenetic distance from each of the test species was calculated in rstudio using APE [139] and the script ‘cophenetic_distance.r’. The precision v.s. evolutionary distance plots and linear regression were conducted in Graphpad prism.

## Supporting information

S1 Fig Experimentally verified and predicted organelle proteins as a percentage of the whole genome. Proteomes of each species from KEGG (Kyoto Encyclopedia of Genes and Genomes) were used as an input for the three algorithm to get proteins predicted as plastid and mitochondria. Their experimental proteomes were taken from organelle proteome databases and literature (see methods). Predicted and experimentally verified plastid (on the left) and mitochondrial (on the right) proteins were plotted as a percentage of all proteins encoded by a given species.
(TIFF)

S2 Fig Number of proteins predicted between plastids and mitochondria. The number of experimentally verified plastid proteins that got predicted as mitochondrial proteins by the three algorithms (on the left) and experimentally verified mitochondrial proteins that got predicted as plastid proteins (on the right).
(TIFF)

S3 Fig Protein clustering and filtering of organelle protein families. All proteins from the four photosynthetic eukaryotes and sorting of protein clusters into plastid (on the left) and mitochondrial family (on the right). Each circle is a protein from a species. In the first step (shown on top), source protein sequences from available species were clustered into protein families (shown at the bottom). If a protein family consisted of an experimentally verified plastid protein (in green, on the left) or a mitochondrial protein (in orange, on the right), the protein family was sorted as a plastid or mitochondrial protein family.
(TIFF)

S4 Fig Chloroplast predicted proteins from TargetP with thylakoid predictions included. Comparison of chloroplast+thylakoid proteins predicted by TargetP2.0 with experimentally localised proteins across species. Each Venn diagram represent data similar to that of Fig 1A, expect now supplemented with ‘thylakoid’ predicted proteins under the category ‘plastid’. The Ven diagrams show an overlap between predicted (left circles) and experimentally verified organelle proteomes (right circles, grey). The underscored numbers in the bottom corners show the total number of predicted (bottom left) and experimentally confirmed proteins (bottom right). The numbers of proteins that overlap (true positives) are provided in the top right corner in bold, while the numbers of non-overlapping ones (false positives) are shown next to each circle. See also the key for the Venn diagrams on the bottom right.
(TIFF)

S5 Fig Taxonomic distribution of TargetP 2.0 training dataset. The targetP2.0 training proteins were downloaded from the original publication and based on their swissprot IDs, their full taxonomy was recovered and number of training proteins per species is plotted here for plastid (a) and mitochondria (b) (with species color coded as per their taxonomy, taxonomy class ‘others’ include: protozoa, insect, nematode, fish, amphibian, amoebozoa, dinoflagellate).
(TIFF)

S6 Fig *Physcomitrium* false negative sorted by BaCeLlo. Experimentally verified *Physcomitrium* organelle proteins that were missed by each algorithm (i.e. the false negative) were used as queries to BaCeLlo to check whether it can sort them correctly. BaCeLlo sorted ca. 50% of them to mitochondria or cytosol, regardless of their verified locations, showing overall affinity for mitochondrial sorting and a lack of reignition for targeting sequence.
(TIFF)

S7 Fig Validation of *Arabidopsis* dual targeting proteins predicted by Ambiguous Targeting Predictor. Dually targeted proteins predicted in *Arabidopsis* by the Ambiguous Targeting Predictor (ATP) compared with mass-spec confirmed dual targeted proteins from *Arabidopsis* shows that ATP missed more than half of *Arabidopsis* dual targeted proteins and predicted ten times more proteins to be dually targeted.
(TIFF)

S8 Fig Dual targeting predictions by cropPAL. cropPAL incorporates a dozen algorithms of which if the majority of algorithm sorts a protein to plastid and mitochondria both, we consider it to be predicted dual targeted. For a given protein, if at least one experimental study experimentally showed plastid and mitochondrial localization, we consider it to be experimentally verified dual targeted protein. If more than one study converge onto plastid and mitochondria, cropPAL labels it as ‘experimental consensus’. Overlap of the three categories (predicted, experimentally verified and experimental consensus) is shown for six species, and they generally underscore overprediction of dual targeting.
(TIFF)

S1 Table List of protein families across species. (XLSX)

S2 Table List of predicted organelle proteins by targetP and orthology approach. (XLSX)

S1 Data Source data for Fig 1A and 1B. (XLSX)

S2 Data Source data for Fig 2A–2D. (XLSX)

S3 Data Source data for Fig 3A–3C. (XLSX)

S4 Data Source data for Fig 4C–4F. (XLSX)

S5 Data Source data for Fig 5A–5D. (XLSX)

S6 Data Source data for S1 Fig. (XLSX)

S7 Data Source data for S2 Fig. (XLSX)

S8 Data Source data for S4 Fig. (XLSX)

S9 Data Source data for S5A and S5B Fig. (XLSX)

S10 Data Source data for S6 Fig. (XLSX)

S11 Data Source data for S7 Fig. (XLSX)

S12 Data Source data for S8 Fig. (XLSX)

## References

[1] Wiedemann N, Pfanner N. Mitochondrial Machineries for Protein Import and Assembly. 2017. doi: 10.1146/annurev-biochem-060815-014352 28301740

[2] Rochaix JD. Chloroplast protein import machinery and quality control. FEBS Journal. John Wiley and Sons Inc; 2022. pp. 6908–6918. doi: 10.1111/febs.16464 35472255 PMC9790281

[3] Gamerdinger M, Deuerling E. Cotranslational sorting and processing of newly synthesized proteins in eukaryotes. Trends Biochem Sci. 2023. 10.1016/j.tibs.2023.10.003 37919225

[4] Gould SB, Garg SG, Martin WF. Bacterial Vesicle Secretion and the Evolutionary Origin of the Eukaryotic Endomembrane System. Trends Microbiol. 2016;24: 525–534. doi: 10.1016/j.tim.2016.03.005 27040918

[5] Raval PK, Garg SG, Gould SB. Endosymbiotic selective pressure at the origin of eukaryotic cell biology. eLife. eLife Sciences Publications Ltd; 2022. doi: 10.7554/eLife.81033 36355038 PMC9648965

[6] Archibald JM. Endosymbiosis and Eukaryotic Cell Evolution. Current Biology. 2015. doi: 10.1016/j.cub.2015.07.055 26439354

[7] Keeling PJ. The Endosymbiotic Origin, Diversification and Fate of Plastids. Philosophical Transactions of the Royal Society B Biological Sciences. 2010. doi: 10.1098/rstb.2009.0103 20124341 PMC2817223

[8] Martin WF, Garg S, Zimorski V. Endosymbiotic theories for eukaryote origin. Philosophical Transactions of the Royal Society B: Biological Sciences. 2015;370. doi: 10.1098/rstb.2014.0330 26323761 PMC4571569

[9] Dacks JB, Field MC. Evolution of the eukaryotic membrane-trafficking system: Origins, tempo and mode. J Cell Sci. 2007;120: 2977–2985. doi: 10.1242/jcs.013250 17715154

[10] Eliáš M. Patterns and Processes in the Evolution of the Eukaryotic Endomembrane System. Molecular Membrane Biology. 2010. doi: 10.3109/09687688.2010.521201 21067450

[11] Elliott L, Moore I, Kirchhelle C. Spatio-temporal control of post-Golgi exocytic trafficking in plants. J Cell Sci. 2020;133. doi: 10.1242/jcs.237065 32102937

[12] Gould SB. Membranes and evolution. Current Biology. 2018;28: R381–R385. doi: 10.1016/j.cub.2018.01.086 29689219

[13] Kelly S. The economics of organellar gene loss and endosymbiotic gene transfer. Genome Biol. 2021;22. doi: 10.1186/s13059-021-02567-w 34930424 PMC8686548

[14] Timmis JN, Ayliff MA, Huang CY, Martin W. Endosymbiotic gene transfer: Organelle genomes forge eukaryotic chromosomes. Nat Rev Genet. 2004;5: 123–135. doi: 10.1038/nrg1271 14735123

[15] Green BR. Chloroplast genomes of photosynthetic eukaryotes. Plant Journal. 2011;66: 34–44. doi: 10.1111/j.1365-313X.2011.04541.x 21443621

[16] Hewitt V, Alcock F, Lithgow T. Minor modifications and major adaptations: The evolution of molecular machines driving mitochondrial protein import. Biochimica et Biophysica Acta—Biomembranes. 2011. pp. 947–954. doi: 10.1016/j.bbamem.2010.07.019 20659421

[17] Hewitt V, Lithgow T, Waller RF. Modifications and innovations in the evolution of mitochondrial protein import pathways. Endosymbiosis. Springer-Verlag Wien; 2014. pp. 19–35. doi: 10.1007/978-3-7091-1303-5_2

[18] Scotti PA, Urbanus ML, Brunner J, De Gier JWL, Von Heijne G, Van Der Does C, et al. YidC, the Escherichia coli homologue of mitochondrial Oxa1p, is a component of the Sec translocase. EMBO Journal. 2000;19: 542–549. doi: 10.1093/emboj/19.4.542 10675323 PMC305592

[19] Hennon SW, Soman R, Zhu L, Dalbey RE. YidC/Alb3/Oxa1 family of insertases. Journal of Biological Chemistry. American Society for Biochemistry and Molecular Biology Inc.; 2015. pp. 14866–14874. doi: 10.1074/jbc.R115.638171 PMC4463434 25947384

[20] Diederichs KA, Buchanan SK, Botos I. Building Better Barrels–β-barrel Biogenesis and Insertion in Bacteria and Mitochondria. Journal of Molecular Biology. Academic Press; 2021. doi: 10.1016/j.jmb.2021.166894 PMC8292188 33639212

[21] Jiang JH, Tong J, Tan KS, Gabriel K. From evolution to Pathogenesis: The link between β-barrel assembly machineries in the outer membrane of mitochondria and Gram-negative bacteria. International Journal of Molecular Sciences. 2012. pp. 8038–8050. doi: 10.3390/ijms13078038 22942688 PMC3430219

[22] Moro F, Fernández-Sáiz V, Slutsky O, Azem A, Muga A. Conformational properties of bacterial DnaK and yeast mitochondrial Hsp70: Role of the divergent C-terminal α-helical subdomain. FEBS Journal. 2005;272: 3184–3196. doi: 10.1111/j.1742-4658.2005.04737.x 15955075

[23] Endow JK, Singhal R, Fernandez DE, Inoue K. Chaperone-assisted post-translational transport of plastidic type i signal peptidase 1. Journal of Biological Chemistry. 2015;290: 28778–28791. doi: 10.1074/jbc.M115.684829 26446787 PMC4661394

[24] Teixeira PF, Glaser E. Processing peptidases in mitochondria and chloroplasts. Biochim Biophys Acta Mol Cell Res. 2013;1833: 360–370. doi: 10.1016/j.bbamcr.2012.03.012 22495024

[25] Ziehe D, Dünschede B, Schünemann D. From bacteria to chloroplasts: Evolution of the chloroplast SRP system. Biological Chemistry. Walter de Gruyter GmbH; 2017. pp. 653–661. doi: 10.1515/hsz-2016-0292 28076289

[26] Schein AI, Kissinger JC, Ungar LH. Chloroplast transit peptide prediction: a peek inside the black box. Nucleic Acids Res. 2001. doi: 10.1093/nar/29.16.e82 11504890 PMC55866

[27] Chen Y, Soman R, Shanmugam SK, Kuhn A, Dalbey RE. The role of the strictly conserved positively charged residue differs among the gram-positive, gram-negative, and chloroplast YidC homologs. Journal of Biological Chemistry. 2014;289: 35656–35667. doi: 10.1074/jbc.M114.595082 25359772 PMC4271247

[28] Day PM, Potter D, Inoue K. Evolution and targeting of omp85 homologs in the chloroplast outer envelope membrane. Front Plant Sci. 2014;5. doi: 10.3389/fpls.2014.00535 25352854 PMC4195282

[29] Knopp M, Garg SG, Handrich M, Gould SB. Major Changes in Plastid Protein Import and the Origin of the Chloroplastida. iScience. 2020;23: 100896. doi: 10.1016/j.isci.2020.100896 32088393 PMC7038456

[30] Paila YD, Richardson LG, Inoue H, Parks ES, Mcmahon J, Inoue K, et al. Multi-functional roles for the polypeptide transport associated domains of Toc75 in chloroplast protein import. Elife. 2016. doi: 10.7554/eLife.12631 26999824 PMC4811774

[31] Richardson LGL, Schnell DJ. Origins, function, and regulation of the TOC-TIC general protein import machinery of plastids. Journal of Experimental Botany. Oxford University Press; 2020. pp. 1226–1238. doi: 10.1093/jxb/erz517 PMC7031061 31730153

[32] Berks BC. The twin-arginine protein translocation pathway. Annual Review of Biochemistry. Annual Reviews Inc.; 2015. pp. 843–864. doi: 10.1146/annurev-biochem-060614-034251 25494301

[33] New CP, Ma Q, Dabney-Smith C. Routing of thylakoid lumen proteins by the chloroplast twin arginine transport pathway. Photosynthesis Research. Springer Netherlands; 2018. pp. 289–301. doi: 10.1007/s11120-018-0567-z 30101370

[34] Robinson C, Bolhuis A. Tat-dependent protein targeting in prokaryotes and chloroplasts. Biochimica et Biophysica Acta—Molecular Cell Research. 2004. pp. 135–147. doi: 10.1016/j.bbamcr.2004.03.010 15546663

[35] Ge C, Spånning E, Glaser E, Wieslander Å. Import determinants of organelle-specific and dual targeting peptides of mitochondria and chloroplasts in arabidopsis thaliana. Mol Plant. 2014;7: 121–136. doi: 10.1093/mp/sst148 24214895

[36] Garg SG, Gould SB. The Role of Charge in Protein Targeting Evolution. Trends Cell Biol. 2016;26: 894–905. doi: 10.1016/j.tcb.2016.07.001 27524662

[37] Bhushan S, Kuhn C, Berglund AK, Roth C, Glaser E. The role of the N-terminal domain of chloroplast targeting peptides in organellar protein import and miss-sorting. FEBS Lett. 2006;580: 3966–3972. doi: 10.1016/j.febslet.2006.06.018 16806197

[38] Lee DW, Lee S, Lee J, Woo S, Razzak MA, Vitale A, et al. Molecular Mechanism of the Specificity of Protein Import into Chloroplasts and Mitochondria in Plant Cells. Mol Plant. 2019;12: 951–966. doi: 10.1016/j.molp.2019.03.003 30890495

[39] Schleiff E, Becker T. Common ground for protein translocation: Access control for mitochondria and chloroplasts. Nat Rev Mol Cell Biol. 2011;12: 48–59. doi: 10.1038/nrm3027 21139638

[40] Carrie C, Small I. A reevaluation of dual-targeting of proteins to mitochondria and chloroplasts. Biochim Biophys Acta Mol Cell Res. 2013;1833: 253–259. doi: 10.1016/j.bbamcr.2012.05.029 22683762

[41] Nesvizhskii AI. A survey of computational methods and error rate estimation procedures for peptide and protein identification in shotgun proteomics. Journal of Proteomics. 2010. pp. 2092–2123. doi: 10.1016/j.jprot.2010.08.009 20816881 PMC2956504

[42] Sun Q, Zybailov B, Majeran W, Friso G, Olinares PDB, van Wijk KJ. PPDB, the Plant Proteomics Database at Cornell. Nucleic Acids Res. 2009;37. doi: 10.1093/nar/gkn654 18832363 PMC2686560

[43] Hooper CM, Castleden IR, Tanz SK, Aryamanesh N, Millar AH. SUBA4: The interactive data analysis centre for Arabidopsis subcellular protein locations. Nucleic Acids Res. 2017;45: D1064–D1074. doi: 10.1093/nar/gkw1041 27899614 PMC5210537

[44] Hooper CM, Castleden IR, Aryamanesh N, Jacoby RP, Millar AH. Finding the Subcellular Location of Barley, Wheat, Rice and Maize Proteins: The Compendium of Crop Proteins with Annotated Locations (cropPAL). Plant Cell Physiology. 2015. doi: 10.1093/pcp/pcv170 26556651

[45] Lisenbee CS, Karnik SK, Trelease RN. Overexpression and mislocalization of a tail-anchored GFP redefines the identity of peroxisomal ER. Traffic. 2003;4: 491–501. doi: 10.1034/j.1600-0854.2003.00107.x 12795694

[46] Jeong K, Kim S, Bandeira N. False discovery rates in spectral identification. BMC Bioinformatics. 2012;13 Suppl 16. doi: 10.1186/1471-2105-13-S16-S2 23176207 PMC3489529

[47] van Wijk KJ, Baginsky S. Plastid proteomics in higher plants: Current state and future goals. Plant Physiol. 2011;155: 1578–1588. doi: 10.1104/pp.111.172932 21350036 PMC3091083

[48] Goodstein DM, Shu S, Howson R, Neupane R, Hayes RD, Fazo J, et al. Phytozome: A comparative platform for green plant genomics. Nucleic Acids Res. 2012;40. doi: 10.1093/nar/gkr944 22110026 PMC3245001

[49] Paysan-Lafosse T, Blum M, Chuguransky S, Grego T, Pinto BL, Salazar GA, et al. InterPro in 2022. Nucleic Acids Res. 2022;51: 418–427. doi: 10.1093/nar/gkac993 36350672 PMC9825450

[50] Nakai K, Kanehisa M. A Knowledge Base for Predicting Protein Localization Sites in Eukaryotic Cells. Genomics. 1992. doi: 10.1016/s0888-7543(05)80111-9 1478671 PMC7134799

[51] Reczko M, Hatzigeorgiou A. Prediction of the subcellular localization of eukaryotic proteins using sequence signals and composition. Proteomics. 2004;4: 1591–1596. doi: 10.1002/pmic.200300769 15174129

[52] Von Heijne G. A new method for predicting signal sequence cleavage sites. Nucleic Acids Res. 1986. doi: 10.1093/nar/14.11.4683 3714490 PMC311474

[53] Bedwell DM, Strobel SA, Yun K, Jongeward GD, Emr SD, Bedwell M, et al. Sequence and Structural Requirements of a Mitochondrial Protein Import Signal Defined by Saturation Cassette Mutagenesis The Saccharomyces cerevisiae Fl-ATPase, subunit precursor contains redundant mitochondrial protein import information at its NH2 terminus (D. Mol Cell Biol. 1989. 10.1128/mcb.9.3.1014-1025.1989 PMC362691 2524645

[54] Nielsen H, Tsirigos KD, Brunak S, von Heijne G. A Brief History of Protein Sorting Prediction. Protein Journal. Springer New York LLC; 2019. pp. 200–216. doi: 10.1007/s10930-019-09838-3 31119599 PMC6589146

[55] Nishikawa K. Correlation of the Amino Acid Composition of a Protein to Its Structural and Biological Characters1. COMMUNICATION J Biochem. 1982. 10.1093/oxfordjournals.jbchem.a133877 7096320

[56] Nishikawa K, Kubota Y, Ooi T. Classification of proteins into groups based on amino acid composition and other characters. I. Angular distribution. J Biochem. 1983;94: 981–995. doi: 10.1093/oxfordjournals.jbchem.a134442 6643432

[57] Nishikawa K, Kubota Y, Ooi T. Classification of proteins into groups based on amino acid composition and other characters. II. Grouping into four types. J Biochem. 1983;94: 997–1007. doi: 10.1093/oxfordjournals.jbchem.a134443 6643433

[58] Mcgeoch DJ. On the predictive recognition of signal peptide sequences. Virus Res. 1985. doi: 10.1016/0168-1702(85)90051-6 3000102

[59] Nakai K, Kanehisa M. Expert system for predicting protein localization sites in gram-negative bacteria. Proteins: Structure, Function, and Bioinformatics. 1991;11: 95–110. doi: 10.1002/prot.340110203 1946347

[60] Walker JM. PSORT: a program for detecting sorting signals in proteins and predicting their subcellular localization. Humana Press; 1998. 10.1016/s0968-0004(98)01336-x 10087920

[61] Nakai K, Horton P. PSORT: a program for detecting sorting signals in proteins and predicting their subcellular localization. Trends Biochem Sci. 1999;24: 34–36. doi: 10.1016/s0968-0004(98)01336-x 10087920

[62] Gardy JL, Spencer C, Wang K, Ester M, Tusnády GE, Simon I, et al. PSORT-B: Improving protein subcellular localization prediction for Gram-negative bacteria. Nucleic Acids Res. 2003;31: 3613–3617. doi: 10.1093/nar/gkg602 12824378 PMC169008

[63] Yu NY, Wagner JR, Laird MR, Melli G, Rey S, Lo R, et al. PSORTb 3.0: Improved protein subcellular localization prediction with refined localization subcategories and predictive capabilities for all prokaryotes. Bioinformatics. 2010;26: 1608–1615. doi: 10.1093/bioinformatics/btq249 20472543 PMC2887053

[64] Horton P, Park KJ, Obayashi T, Fujita N, Harada H, Adams-Collier CJ, et al. WoLF PSORT: Protein localization predictor. Nucleic Acids Res. 2007;35. doi: 10.1093/nar/gkm259 17517783 PMC1933216

[65] Horton PA, Park KA, Obayashi TB, Nakai KC. Protein subcellular localisation prediction using WOLF PSORT. Conference: Proceedings of 4th Asia-Pacific Bioinformatics Conference. 13–16 February 2006, Taipei, Taiwan; 2005. doi: 10.1142/9781860947292_0007

[66] Armenteros JJA, Salvatore M, Emanuelsson O, Winther O, Von Heijne G, Elofsson A, et al. Detecting sequence signals in targeting peptides using deep learning. Life Sci Alliance. 2019;2: 1–14. doi: 10.26508/lsa.201900429 31570514 PMC6769257

[67] Blake JA, Dolan M, Drabkin H, Hill DP, Ni L, Sitnikov D, et al. Gene ontology annotations and resources. Nucleic Acids Res. 2013;41. doi: 10.1093/nar/gks1050 23161678 PMC3531070

[68] Hooper CM, Castleden IR, Aryamanesh N, Jacoby RP, Millar AH. Finding the Subcellular Location of Barley, Wheat, Rice and Maize Proteins: The Compendium of Crop Proteins with Annotated Locations (cropPAL). Plant Cell Physiol. 2015;57(1). doi: 10.1093/pcp/pcv170 26556651

[69] Hooper CM, Tanz SK, Castleden IR, Vacher MA, Small ID, Millar AH. SUBAcon: A consensus algorithm for unifying the subcellular localization data of the Arabidopsis proteome. Bioinformatics. 2014;30: 3356–3364. doi: 10.1093/bioinformatics/btu550 25150248

[70] Hooper CM, Castleden IR, Aryamanesh N, Black K, Grasso S V., Millar AH. CropPAL for discovering divergence in protein subcellular location in crops to support strategies for molecular crop breeding. Plant Journal. 2020;104: 812–827. doi: 10.1111/tpj.14961 32780488

[71] Boval M, Dixon RM. The importance of grasslands for animal production and other functions: A review on management and methodological progress in the tropics. Animal. 2012. pp. 748–762. doi: 10.1017/S1751731112000304 22558923

[72] José J, Karlusich P, Ibarbalz FM, Bowler C. Phytoplankton in the Tara Ocean. 2019. doi: 10.1146/annurev-marine-010419 31899671

[73] Linder HP, Lehmann CER, Archibald S, Osborne CP, Richardson DM. Global grass (Poaceae) success underpinned by traits facilitating colonization, persistence and habitat transformation. Biological Reviews. 2018;93: 1125–1144. doi: 10.1111/brv.12388 29230921

[74] Frangedakis E, Marron AO, Waller M, Neubauer A, Tse SW, Yue Y, et al. What can hornworts teach us? Frontiers in Plant Science. Frontiers Media S.A.; 2023. doi: 10.3389/fpls.2023.1108027 36968370 PMC10030945

[75] Li FW, Nishiyama T, Waller M, Frangedakis E, Keller J, Li Z, et al. Anthoceros genomes illuminate the origin of land plants and the unique biology of hornworts. Nat Plants. 2020;6: 259–272. doi: 10.1038/s41477-020-0618-2 32170292 PMC8075897

[76] Rensing SA, Goffinet B, Meyberg R, Wu SZ, Bezanilla M. The moss physcomitrium (Physcomitrella) patens: A model organism for non-seed plants. Plant Cell. 2020;32: 1361–1376. doi: 10.1105/tpc.19.00828 32152187 PMC7203925

[77] Lang D, Ullrich KK, Murat F, Fuchs J, Jenkins J, Haas FB, et al. The Physcomitrella patens chromosome-scale assembly reveals moss genome structure and evolution. Plant Journal. 2018;93: 515–533. doi: 10.1111/tpj.13801 29237241

[78] Raval PK, MacLeod AI, Gould SB. A molecular atlas of plastid and mitochondrial proteins reveals organellar remodeling during plant evolutionary transitions from algae to angiosperms. PLoS Biol. 2024;22. doi: 10.1371/journal.pbio.3002608 38713727 PMC11135702

[79] Christian RW, Hewitt SL, Roalson EH, Dhingra A. Genome-Scale Characterization of Predicted Plastid-Targeted Proteomes in Higher Plants. Sci Rep. 2020;10: 1–22. doi: 10.1038/s41598-020-64670-5 32427841 PMC7237471

[80] de Vries J, Stanton A, Archibald JM, Gould SB. Streptophyte Terrestrialization in Light of Plastid Evolution. Trends Plant Sci. 2016;21: 467–476. doi: 10.1016/j.tplants.2016.01.021 26895731

[81] Schreiber M, Rensing SA, Gould SB. The greening ashore. Trends in Plant Science. Elsevier Ltd; 2022. pp. 847–857. doi: 10.1016/j.tplants.2022.05.005 35739050

[82] Emms DM, Kelly S. OrthoFinder: Phylogenetic orthology inference for comparative genomics. Genome Biol. 2019;20: 1–14. doi: 10.1186/s13059-019-1832-y 31727128 PMC6857279

[83] Heinnickel ML, Grossman AR. The GreenCut: Re-evaluation of physiological role of previously studied proteins and potential novel protein functions. Photosynth Res. 2013;116: 427–436. doi: 10.1007/s11120-013-9882-6 23873414

[84] Schaeffer SM, Christian R, Castro-Velasquez N, Hyden B, Lynch-Holm V, Dhingra A. Comparative ultrastructure of fruit plastids in three genetically diverse genotypes of apple (Malus × domestica Borkh.) during development. Plant Cell Rep. 2017;36: 1627–1640. doi: 10.1007/s00299-017-2179-z 28698906 PMC5693628

[85] Richly E, Leister D. An improved prediction of chloroplast proteins reveals diversities and commonalities in the chloroplast proteomes of Arabidopsis and rice. Gene. 2004;329: 11–16. doi: 10.1016/j.gene.2004.01.008 15033524

[86] Li L, Yuan H. Chromoplast biogenesis and carotenoid accumulation. Archives of Biochemistry and Biophysics. 2013. pp. 102–109. doi: 10.1016/j.abb.2013.07.002 23851381

[87] Choi H, Yi T, Ha SH. Diversity of Plastid Types and Their Interconversions. Frontiers in Plant Science. Frontiers Media S.A.; 2021. doi: 10.3389/fpls.2021.692024 34220916 PMC8248682

[88] Kleffmann T, Hirsch-Hoffmann M, Gruissem W, Baginsky S. plprot: A comprehensive proteome database for different plastid types. Plant Cell Physiol. 2006;47: 432–436. doi: 10.1093/pcp/pcj005 16418230

[89] Breckels LM, Holden SB, Wojnar D, Mulvey CM, Christoforou A, Groen A, et al. Learning from Heterogeneous Data Sources: An Application in Spatial Proteomics. PLoS Comput Biol. 2016;12. doi: 10.1371/journal.pcbi.1004920 27175778 PMC4866734

[90] Murcha MW, Kmiec B, Kubiszewski-Jakubiak S, Teixeira PF, Glaser E, Whelan J. Protein import into plant mitochondria: signals, machinery, processing, and regulation. J Exp Bot. 2014;65: 6301–6335. doi: 10.1093/jxb/eru399 25324401

[91] Murcha MW, Wang Y, Narsai R, Whelan J. The plant mitochondrial protein import apparatus—The differences make it interesting. Biochimica et Biophysica Acta (BBA)—General Subjects. 2014;1840: 1233–1245. doi: 10.1016/j.bbagen.2013.09.026 24080405

[92] Heidorn-Czarna M, Maziak A, Janska H. Protein Processing in Plant Mitochondria Compared to Yeast and Mammals. Front Plant Sci. 2022;13. doi: 10.3389/fpls.2022.824080 35185991 PMC8847149

[93] Carrie C, Murcha MW, Whelan J. An in silico analysis of the mitochondrial protein import apparatus of plants. BMC Plant Biol. 2010;10: 249. doi: 10.1186/1471-2229-10-249 21078193 PMC3095331

[94] Lister R, Carrie C, Duncan O, Ho LHM, Howell KA, Murcha MW, et al. Functional definition of outer membrane proteins involved in preprotein import into mitochondria. Plant Cell. 2007;19: 3739–3759. doi: 10.1105/tpc.107.050534 17981999 PMC2174869

[95] Perry AJ, Hulett JM, Likić VA, Lithgow T, Gooley PR. Convergent Evolution of Receptors for Protein Import into Mitochondria. Current Biology. 2006;16: 221–229. doi: 10.1016/j.cub.2005.12.034 16461275

[96] Rimmer KA, Foo JH, Ng A, Petrie EJ, Shilling PJ, Perry AJ, et al. Recognition of mitochondrial targeting sequences by the import receptors Tom20 and Tom22. J Mol Biol. 2011;405: 804–818. doi: 10.1016/j.jmb.2010.11.017 21087612

[97] Chew O, Lister R, Qbadou S, Heazlewood JL, Soll J, Schleiff E, et al. A plant outer mitochondrial membrane protein with high amino acid sequence identity to a chloroplast protein import receptor. FEBS Lett. 2004;557: 109–114. doi: 10.1016/s0014-5793(03)01457-1 14741350

[98] Huang S, Taylor NL, Whelan J, Millar AH. Refining the Definition of Plant Mitochondrial Presequences through Analysis of Sorting Signals, N-Terminal Modifications, and Cleavage Motifs. Plant Physiol. 2009;150: 1272. doi: 10.1104/pp.109.137885 19474214 PMC2705053

[99] Patron NJ, Waller RF. Transit peptide diversity and divergence: A global analysis of plastid targeting signals. BioEssays. 2007;29: 1048–1058. doi: 10.1002/bies.20638 17876808

[100] Fuss J, Liegmann O, Krause K, Rensing SA. Green Targeting Predictor and Ambiguous Targeting Predictor 2: the pitfalls of plant protein targeting prediction and of transient protein expression in heterologous systems. New Phytologist. 2013;200: 1022–1033. doi: 10.1111/nph.12433 23915300

[101] Huang S, Taylor NL, Whelan J, Millar AH. Refining the definition of plant mitochondrial presequences through analysis of sorting signals, n-terminal modifications, and cleavage motifs. Plant Physiol. 2009;150: 1272–1285. doi: 10.1104/pp.109.137885 19474214 PMC2705053

[102] Zhang X-P, Glaser E. Interaction of plant mitochondrial and chloroplast signal peptides with the Hsp70 molecular chaperone. Trends Plant Sci. 2002;7: 14–21. doi: 10.1016/s1360-1385(01)02180-x 11804822

[103] Razzak MA, Lee DW, Yoo YJ, Hwang I. Evolution of rubisco complex small subunit transit peptides from algae to plants. Sci Rep. 2017;7. doi: 10.1038/s41598-017-09473-x 28839179 PMC5571161

[104] Sáiz-Bonilla M, Martín Merchán A, Pallás V, Navarro JA. Molecular characterization, targeting and expression analysis of chloroplast and mitochondrion protein import components in Nicotiana benthamiana. Front Plant Sci. 2022;13: 1040688. doi: 10.3389/fpls.2022.1040688 36388587 PMC9643744

[105] Schnell DJ. The TOC GTPase Receptors: Regulators of the Fidelity, Specificity and Substrate Profiles of the General Protein Import Machinery of Chloroplasts. Protein J. 2019;38. doi: 10.1007/s10930-019-09846-3 31201619 PMC6589150

[106] Yan J, Campbell JH, Glick BR, Smith MD, Liang Y. Molecular characterization and expression analysis of chloroplast protein import components in tomato (Solanum lycopersicum). PLoS One. 2014;9. doi: 10.1371/journal.pone.0095088 24751891 PMC3994019

[107] Paul P, Simm S, Blaumeiser A, Scharf KD, Fragkostefanakis S, Mirus O, et al. The protein translocation systems in plants—composition and variability on the example of Solanum lycopersicum. BMC Genomics. 2013;14: 1–16. doi: 10.1186/1471-2164-14-189/FIGURES/4 23506162 PMC3610429

[108] Stengel A, Benz JP, Buchanan BB, Soll J, Bölter B. Preprotein import into chloroplasts via the Toc and Tic complexes is regulated by redox signals in Pisum sativum. Mol Plant. 2009;2: 1181–1197. doi: 10.1093/mp/ssp043 19995724

[109] Pierleoni A, Martelli PL, Fariselli P, Casadio R. BaCelLo: A balanced subcellular localization predictor. Bioinformatics. Oxford University Press; 2006. doi: 10.1093/bioinformatics/btl222 16873501

[110] Elo A, Lyznik A, Gonzalez DO, Kachman SD, Mackenzie SA. Nuclear genes that encode mitochondrial proteins for DNA and RNA metabolism are clustered in the Arabidopsis genome. Plant Cell. 2003;15: 1619–1631. doi: 10.1105/tpc.010009 12837951 PMC165405

[111] Carrie C, Giraud E, Whelan J. Protein transport in organelles: Dual targeting of proteins to mitochondria and chloroplasts. FEBS J. 2009;276: 1187–1195. doi: 10.1111/j.1742-4658.2009.06876.x 19187233

[112] Martin W. Evolutionary origins of metabolic compartmentalization in eukaryotes. Philosophical Transactions of the Royal Society B: Biological Sciences. 2010;365: 847–855. doi: 10.1098/RSTB.2009.0252 20124349 PMC2817231

[113] Xu L, Carrie C, Law SR, Murcha MW, Whelan J. Acquisition, Conservation, and Loss of Dual-Targeted Proteins in Land Plants. Plant Physiol. 2013;161: 644. doi: 10.1104/pp.112.210997 23257241 PMC3561010

[114] Morgante C V., Rodrigues RAO, Marbach PAS, Borgonovi CM, Moura DS, Silva-Filho MC. Conservation of dual-targeted proteins in Arabidopsis and rice points to a similar pattern of gene-family evolution. Molecular Genetics and Genomics. 2009;281: 525–538. doi: 10.1007/S00438-009-0429-7/FIGURES/2 19214577

[115] Burak E, Yogev O, Sheffer S, Schueler-Furman O, Pines O. Evolving dual targeting of a prokaryotic protein in yeast. Mol Biol Evol. 2013;30: 1563–1573. doi: 10.1093/molbev/mst039 23462316

[116] Tardif M, Atteia A, Specht M, Cogne G, Rolland N, Brugière S, et al. Predalgo: A new subcellular localization prediction tool dedicated to green algae. Molecular Biology and Evolution. 2012. pp. 3625–3639. doi: 10.1093/molbev/mss178 22826458

[117] Cleary SP, Tan FC, Nakrieko KA, Thompson SJ, Mullineaux PM, Creissen GP, et al. Isolated Plant Mitochondria Import Chloroplast Precursor Proteinsin Vitro with the Same Efficiency as Chloroplasts. Journal of Biological Chemistry. 2002;277: 5562–5569. doi: 10.1074/jbc.M106532200 11733507

[118] Chew O, Rudhe C, Glaser E, Whelan J. Characterization of the targeting signal of dual-targeted pea glutathione reductase. Plant Mol Biol. 2003;53: 341–356. doi: 10.1023/b:plan.0000006939.87660.4f 14750523

[119] Lister R, Chew O, Rudhe C, Lee MN, Whelan J. Arabidopsis thaliana ferrochelatase-I and -II are not imported into Arabidopsis mitochondria. FEBS Lett. 2001;506: 291–295. doi: 10.1016/s0014-5793(01)02925-8 11602264

[120] Hurt EC, Soltanifar N, Goldschmidt-Clermont M, Rochaix J-D, Schatz G. The cleavable pre-sequence of an imported chloroplast protein directs attached polypeptides into yeast mitochondria. EMBO J. 1986;5: 1343–1350. doi: 10.1002/j.1460-2075.1986.tb04365.x 16453686 PMC1166946

[121] Mitschke J, Fuss J, Blum T, Höglund A, Reski R, Kohlbacher O, et al. Prediction of dual protein targeting to plant organelles: Methods. New Phytologist. 2009;183: 224–236. doi: 10.1111/j.1469-8137.2009.02832.x 19368670

[122] Garg S, Stölting J, Zimorski V, Rada P, Tachezy J, Martin WF, et al. Conservation of transit peptide-Independent protein import into the mitochondrial and hydrogenosomal matrix. Genome Biol Evol. 2015;7: 2716–2726. doi: 10.1093/gbe/evv175 26338186 PMC4607531

[123] Burstein D, Gould SB, Zimorski V, Kloesges T, Kiosse F, Major P, et al. A machine learning approach to identify hydrogenosomal proteins in trichomonas vaginalis. Eukaryot Cell. 2012;11: 217–228. doi: 10.1128/EC.05225-11 22140228 PMC3272890

[124] Wang L, Patena W, Van Baalen KA, Xie Y, Singer ER, Gavrilenko S, et al. A chloroplast protein atlas reveals punctate structures and spatial organization of biosynthetic pathways. Cell. 2023;186: 3499–3518.e14. doi: 10.1016/j.cell.2023.06.008 37437571

[125] Gruber A, Rocap G, Kroth PG, Armbrust EV, Mock T. Plastid proteome prediction for diatoms and other algae with secondary plastids of the red lineage. Plant Journal. 2015;81: 519–528. doi: 10.1111/tpj.12734 25438865 PMC4329603

[126] Mulvey CM, Breckels LM, Geladaki A, Britovšek NK, Nightingale DJH, Christoforou A, et al. Using hyperLOPIT to perform high-resolution mapping of the spatial proteome. Nat Protoc. 2017;12: 1110–1135. doi: 10.1038/nprot.2017.026 28471460

[127] Geladaki A, Kočevar Britovšek N, Breckels LM, Smith TS, Vennard OL, Mulvey CM, et al. Combining LOPIT with differential ultracentrifugation for high-resolution spatial proteomics. Nat Commun. 2019;10. doi: 10.1038/s41467-018-08191-w 30659192 PMC6338729

[128] Wang S, Li L, Li H, Sahu SK, Wang H, Xu Y, et al. Genomes of early-diverging streptophyte algae shed light on plant terrestrialization. Nat Plants. 2020;6: 95–106. doi: 10.1038/s41477-019-0560-3 31844283 PMC7027972

[129] Cheng S, Xian W, Fu Y, Marin B, Keller J, Wu T, et al. Genomes of Subaerial Zygnematophyceae Provide Insights into Land Plant Evolution. Cell. 2019;179: 1057–1067.e14. doi: 10.1016/j.cell.2019.10.019 31730849

[130] Bowman JL, Kohchi T, Yamato KT, Jenkins J, Shu S, Ishizaki K, et al. Insights into Land Plant Evolution Garnered from the Marchantia polymorpha Genome. Cell. 2017;171: 287–304.e15. doi: 10.1016/j.cell.2017.09.030 28985561

[131] Nishiyama T, Sakayama H, de Vries J, Buschmann H, Saint-Marcoux D, Ullrich KK, et al. The Chara Genome: Secondary Complexity and Implications for Plant Terrestrialization. Cell. 2018;174: 448–464.e24. doi: 10.1016/j.cell.2018.06.033 30007417

[132] Hori K, Maruyama F, Fujisawa T, Togashi T, Yamamoto N, Seo M, et al. Klebsormidium flaccidum genome reveals primary factors for plant terrestrial adaptation. Nat Commun. 2014;5. doi: 10.1038/ncomms4978 24865297 PMC4052687

[133] Bordin N, Dallago C, Heinzinger M, Kim S, Littmann M, Rauer C, et al. Novel machine learning approaches revolutionize protein knowledge. Trends in Biochemical Sciences. Elsevier Ltd; 2023. pp. 345–359. doi: 10.1016/j.tibs.2022.11.001 PMC10570143 36504138

[134] Hesami M, Alizadeh M, Jones AMP, Torkamaneh D. Machine learning: its challenges and opportunities in plant system biology. Applied Microbiology and Biotechnology. Springer Science and Business Media Deutschland GmbH; 2022. pp. 3507–3530. doi: 10.1007/s00253-022-11963-6 35575915

[135] Kanehisa M, Furumichi M, Sato Y, Kawashima M, Ishiguro-Watanabe M. KEGG for taxonomy-based analysis of pathways and genomes. Nucleic Acids Res. 2023;51: D587–D592. doi: 10.1093/nar/gkac963 36300620 PMC9825424

[136] Atteia A, Adrait A, Brugire S, Tardif M, Van Lis R, Deusch O, et al. A proteomic survey of chlamydomonas reinhardtii mitochondria sheds new light on the metabolic plasticity of the organelle and on the nature of the α-proteobacterial mitochondrial ancestor. Mol Biol Evol. 2009;26: 1533–1548. doi: 10.1093/molbev/msp068 19349646

[137] Terashima M, Specht M, Hippler M. The chloroplast proteome: A survey from the Chlamydomonas reinhardtii perspective with a focus on distinctive features. Current Genetics. 2011. pp. 151–168. doi: 10.1007/s00294-011-0339-1 21533645

[138] Mueller SJ, Lang D, Hoernstein SNW, Lang EGE, Schuessele C, Schmidt A, et al. Quantitative analysis of the mitochondrial and plastid proteomes of the moss Physcomitrella patens reveals protein macrocompartmentation and microcompartmentation. Plant Physiol. 2014;164: 2081–2095. doi: 10.1104/pp.114.235754 24515833 PMC3982764

[139] Paradis E, Claude J, Strimmer K. APE: Analyses of phylogenetics and evolution in R language. Bioinformatics. 2004;20: 289–290. doi: 10.1093/bioinformatics/btg412 14734327

