# Supplementary figures and images for: Reliability of plastid and mitochondrial localisation prediction declines rapidly with the evolutionary distance to the training set increasing
Source: PLoS Comput Biol. 2024 Nov 11;20(11):e1012575. doi: 10.1371/journal.pcbi.1012575 (PMC11581415; doi:10.1371/journal.pcbi.1012575)

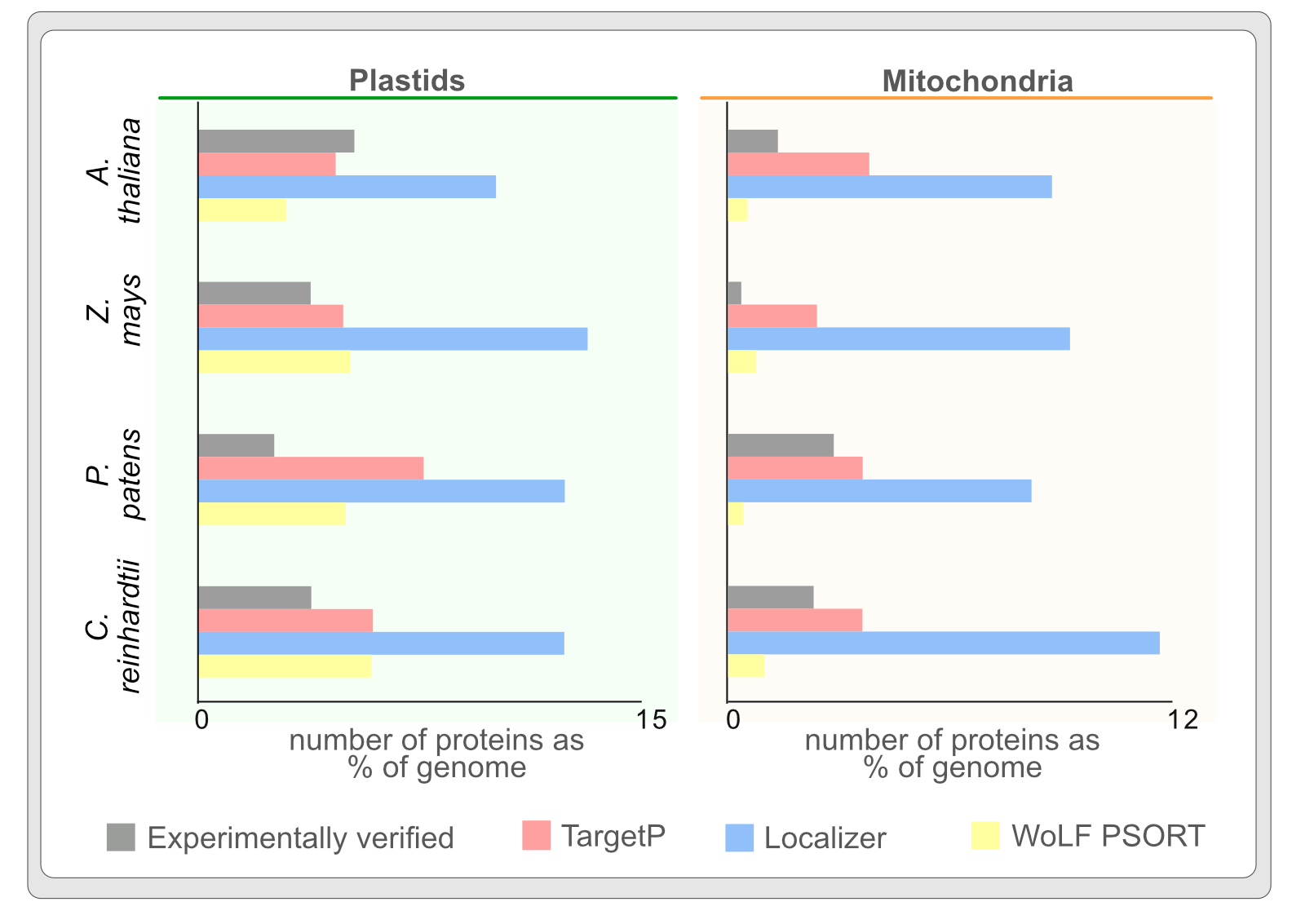

Supplement: S1 Fig — Proteomes of each species from KEGG (Kyoto Encyclopedia of Genes and Genomes) were used as an input for the three algorithm to get proteins predicted as plastid and mitochondria. Their experimental proteomes were taken from organelle proteome databases and literature (see methods). Predicted and experimentally verified plastid (on the left) and mitochondrial (on the right) proteins were plotted as a percentage of all proteins encoded by a given species. (TIFF) [file pcbi.1012575.s001.tiff]

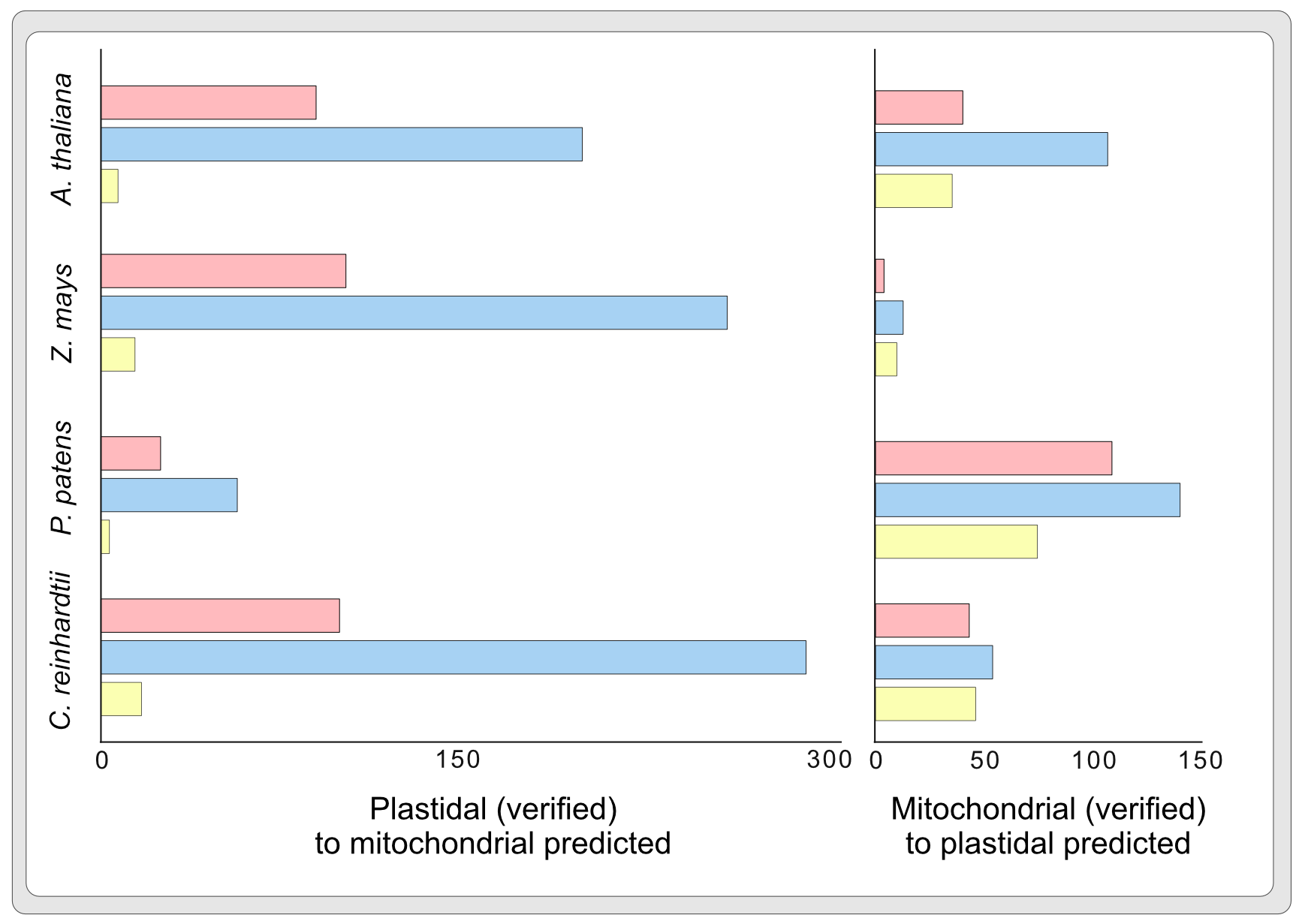

Supplement: S2 Fig — The number of experimentally verified plastid proteins that got predicted as mitochondrial proteins by the three algorithms (on the left) and experimentally verified mitochondrial proteins that got predicted as plastid proteins (on the right). (TIFF) [file pcbi.1012575.s002.tiff]

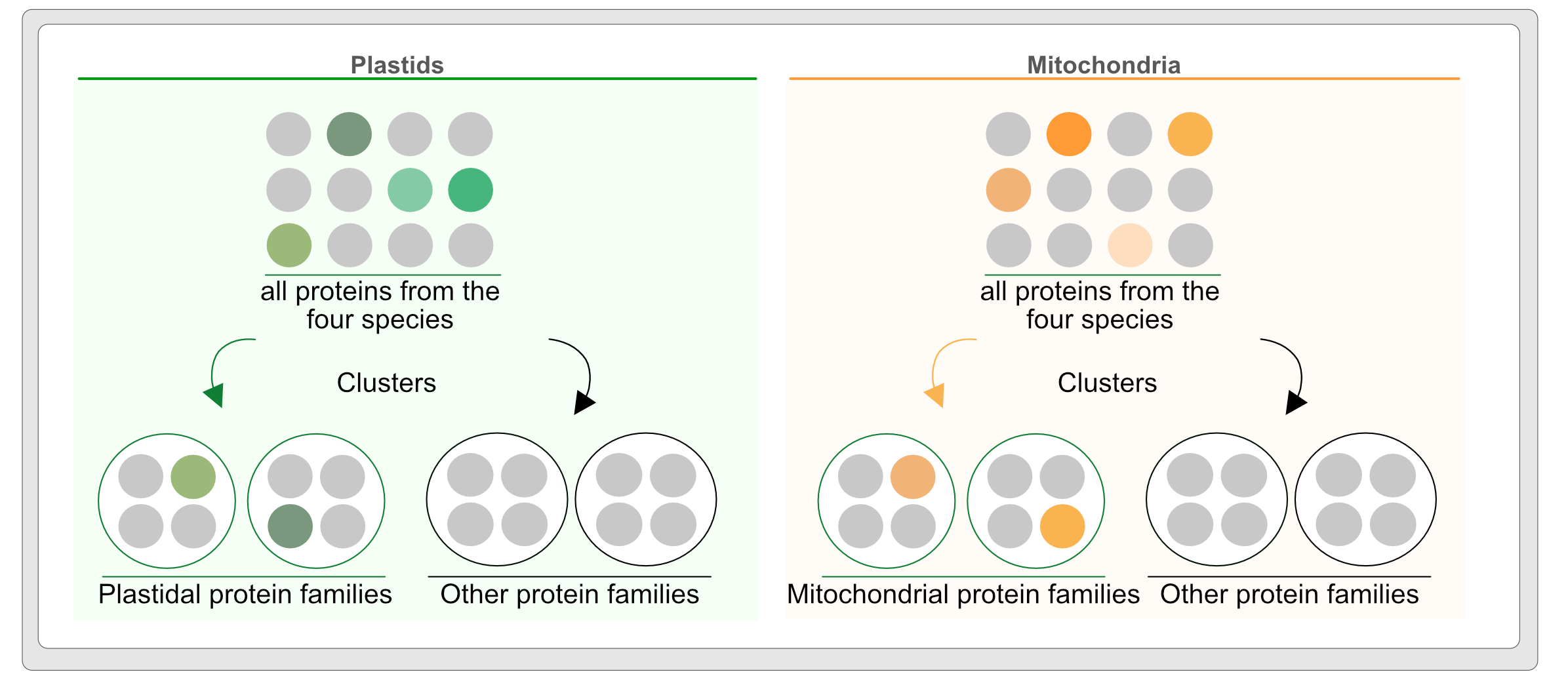

Supplement: S3 Fig — All proteins from the four photosynthetic eukaryotes and sorting of protein clusters into plastid (on the left) and mitochondrial family (on the right). Each circle is a protein from a species. In the first step (shown on top), source protein sequences from available species were clustered into protein families (shown at the bottom). If a protein family consisted of an experimentally verified plastid protein (in green, on the left) or a mitochondrial protein (in orange, on the right), the protein family was sorted as a plastid or mitochondrial protein family. (TIFF) [file pcbi.1012575.s003.tiff]

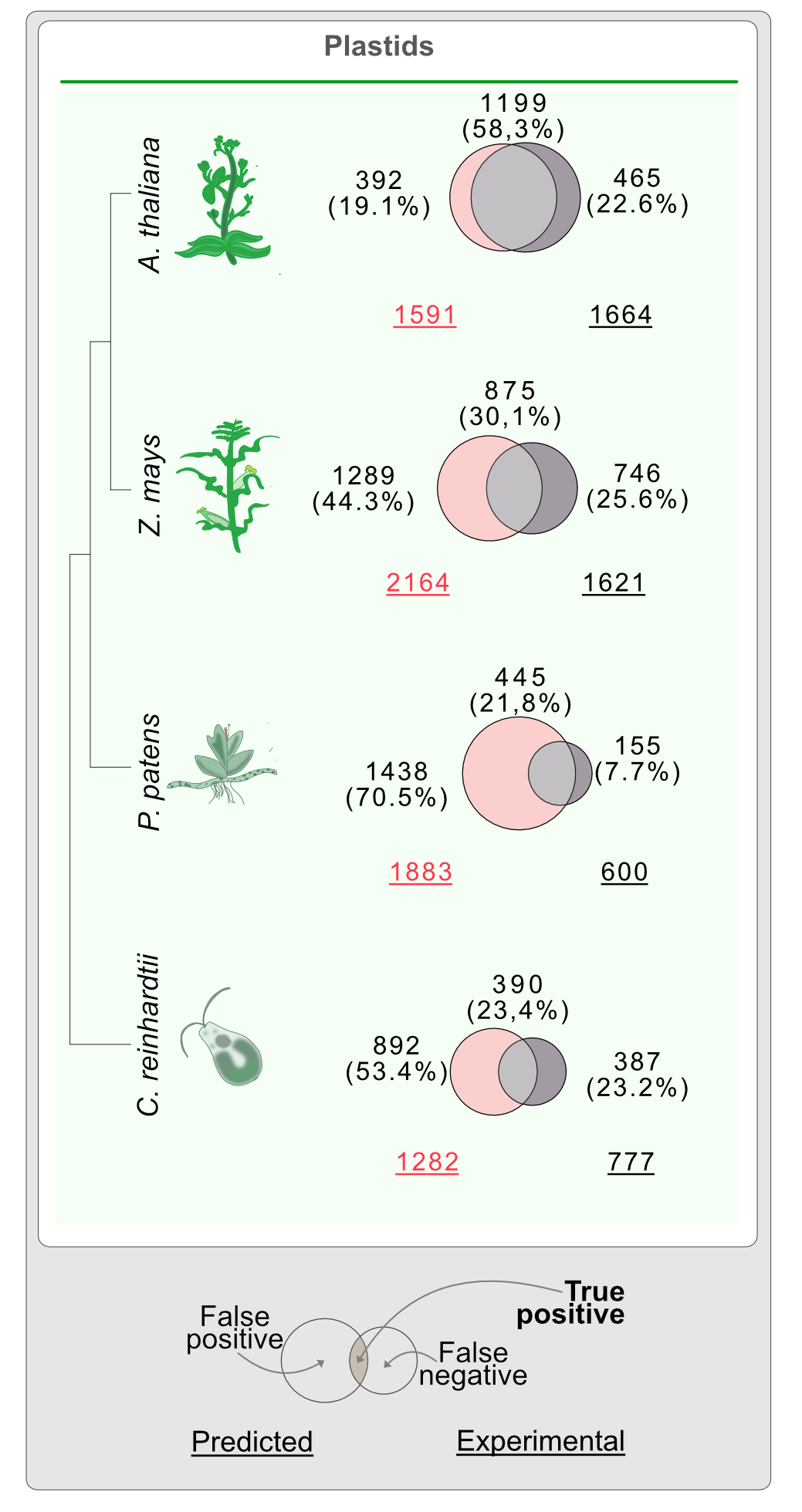

Supplement: S4 Fig — Comparison of chloroplast+thylakoid proteins predicted by TargetP2.0 with experimentally localised proteins across species. Each Venn diagram represent data similar to that of Fig 1A, expect now supplemented with ‘thylakoid’ predicted proteins under the category ‘plastid’. The Ven diagrams show an overlap between predicted (left circles) and experimentally verified organelle proteomes (right circles, grey). The underscored numbers in the bottom corners show the total number of predicted (bottom left) and experimentally confirmed proteins (bottom right). The numbers of proteins that overlap (true positives) are provided in the top right corner in bold, while the numbers of non-overlapping ones (false positives) are shown next to each circle. See also the key for the Venn diagrams on the bottom right. (TIFF) [file pcbi.1012575.s004.tiff]

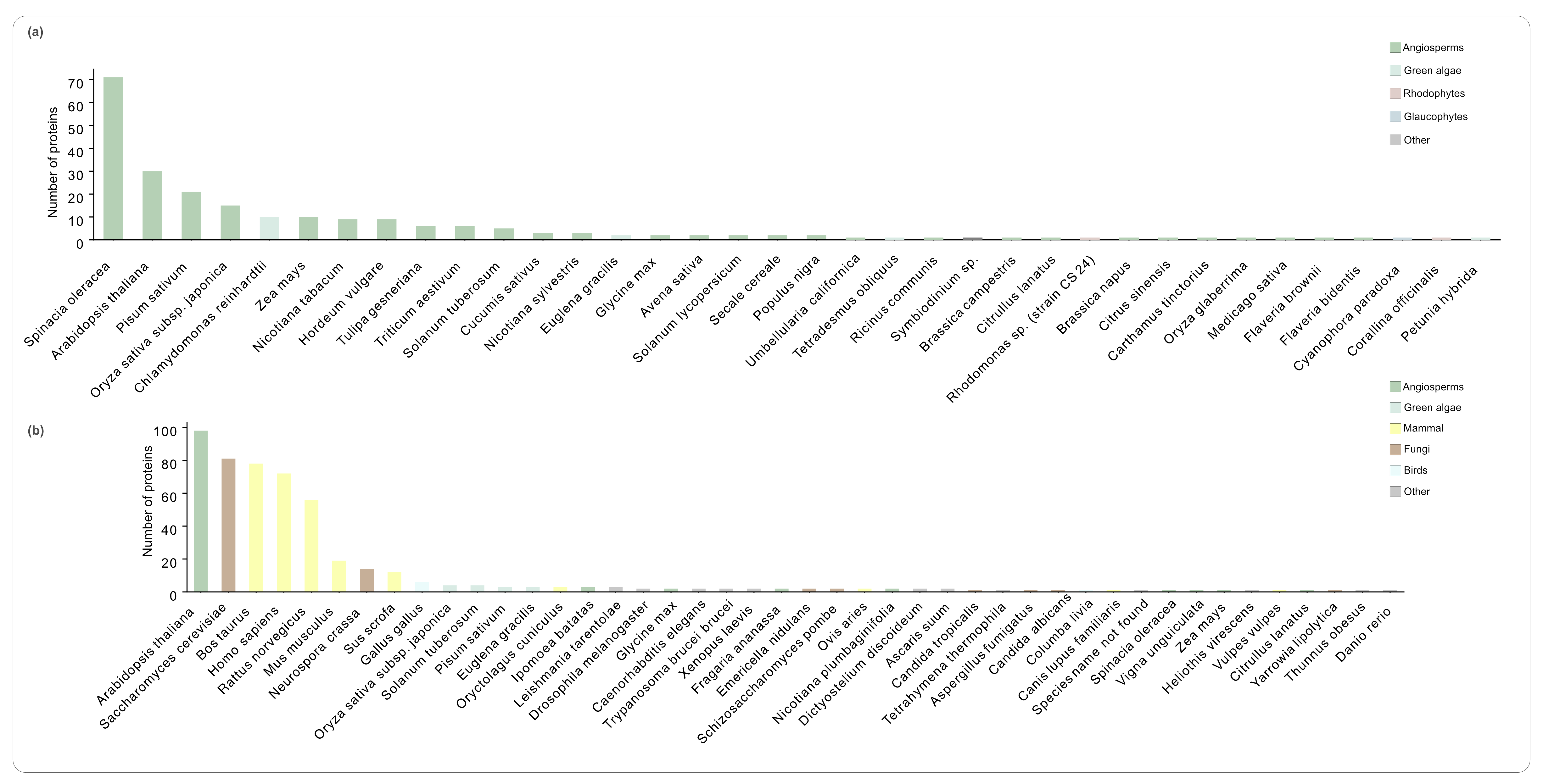

Supplement: S5 Fig — The targetP2.0 training proteins were downloaded from the original publication and based on their swissprot IDs, their full taxonomy was recovered and number of training proteins per species is plotted here for plastid (a) and mitochondria (b) (with species color coded as per their taxonomy, taxonomy class ‘others’ include: protozoa, insect, nematode, fish, amphibian, amoebozoa, dinoflagellate). (TIFF) [file pcbi.1012575.s005.tiff]

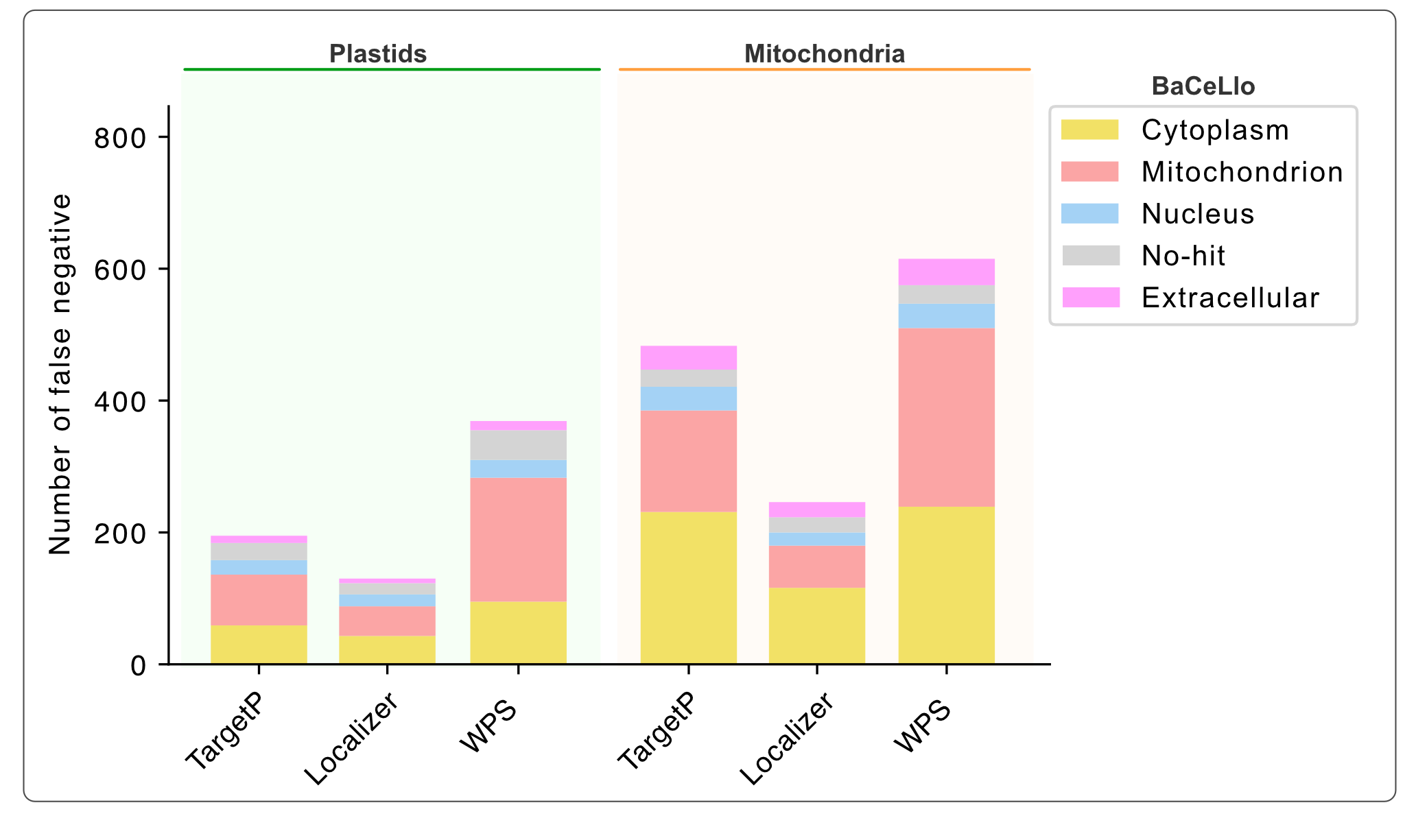

Supplement: S6 Fig — Experimentally verified Physcomitrium organelle proteins that were missed by each algorithm (i.e. the false negative) were used as queries to BaCeLlo to check whether it can sort them correctly. BaCeLlo sorted ca. 50% of them to mitochondria or cytosol, regardless of their verified locations, showing overall affinity for mitochondrial sorting and a lack of reignition for targeting sequence. (TIFF) [file pcbi.1012575.s006.tiff]

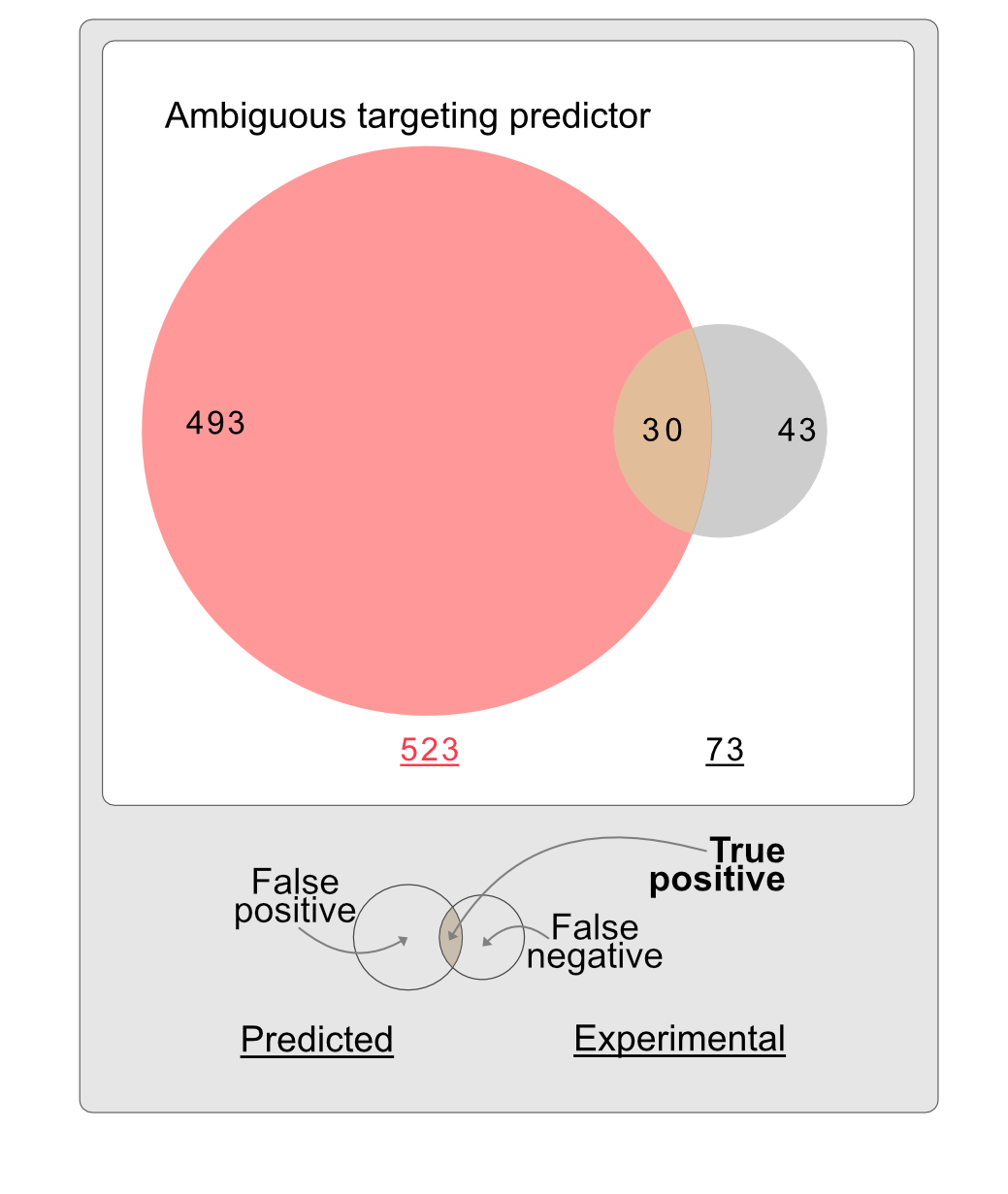

Supplement: S7 Fig — Dually targeted proteins predicted in Arabidopsis by the Ambiguous Targeting Predictor (ATP) compared with mass-spec confirmed dual targeted proteins from Arabidopsis shows that ATP missed more than half of Arabidopsis dual targeted proteins and predicted ten times more proteins to be dually targeted. (TIFF) [file pcbi.1012575.s007.tiff]

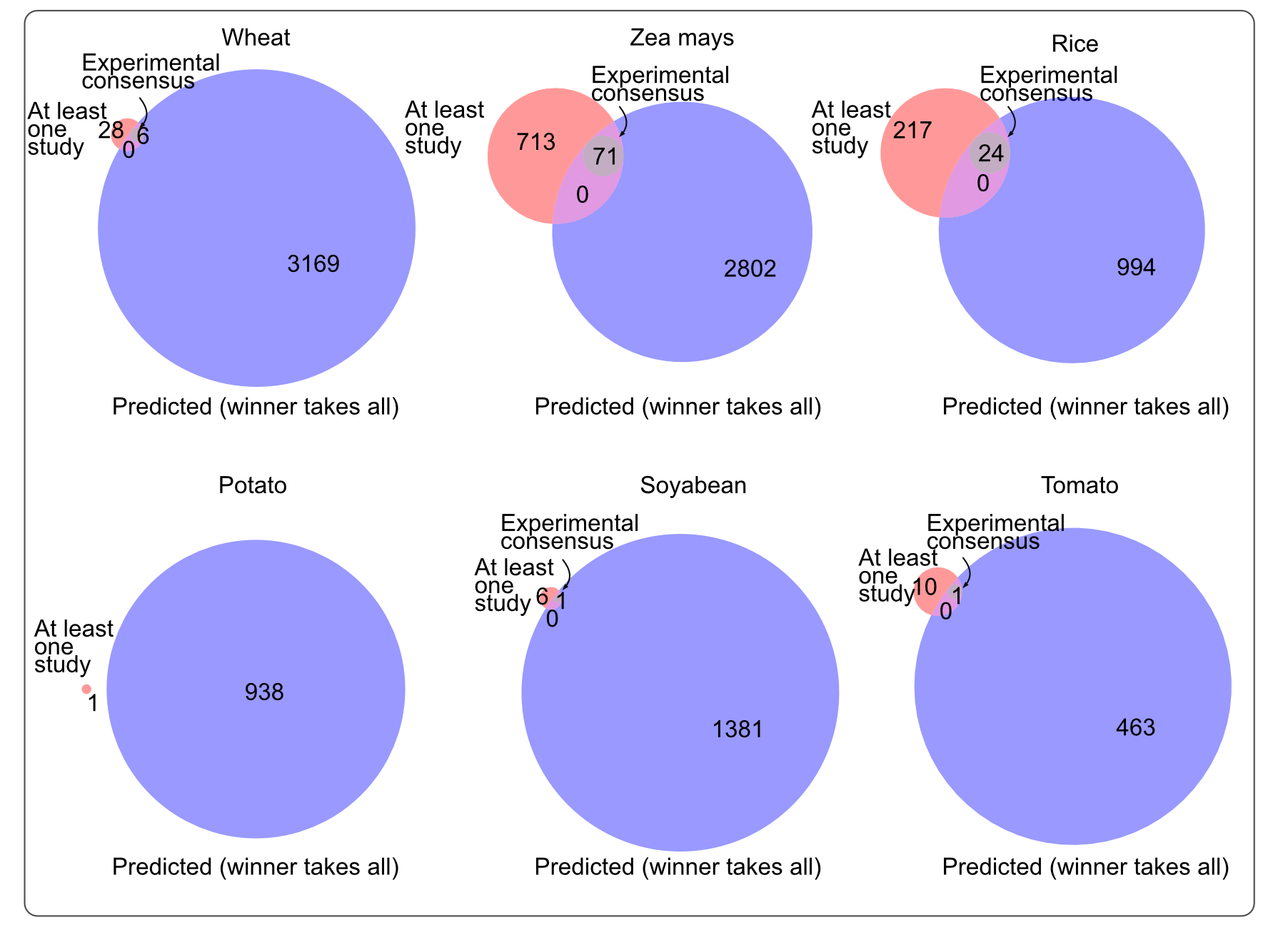

Supplement: S8 Fig — cropPAL incorporates a dozen algorithms of which if the majority of algorithm sorts a protein to plastid and mitochondria both, we consider it to be predicted dual targeted. For a given protein, if at least one experimental study experimentally showed plastid and mitochondrial localization, we consider it to be experimentally verified dual targeted protein. If more than one study converge onto plastid and mitochondria, cropPAL labels it as ‘experimental consensus’. Overlap of the three categories (predicted, experimentally verified and experimental consensus) is shown for six species, and they generally underscore overprediction of dual targeting. (TIFF) [file pcbi.1012575.s008.tiff]
