# Supplementary material for: Reliability of plastid and mitochondrial localisation prediction declines rapidly with the evolutionary distance to the training set increasing
Source: PLoS Comput Biol. 2024 Nov 11;20(11):e1012575. doi: 10.1371/journal.pcbi.1012575 (PMC11581415; doi:10.1371/journal.pcbi.1012575)
Supplement: Attachment — Submitted filename: Response to Reviewers.pdf [file pcbi.1012575.s023.pdf]

Reviewer 1

Reviewer #1: In this manuscript, the authors reported their benchmark study on the prediction accuracy of three widely used algorithms for localization into plant (or photosynthetic eukaryotes') plastids and mitochondria using experimentally determined proteome data supplemented by homology-based data. Their results show that the performance of these tools is not practically reliable and that the accuracy becomes even worse in plant species with more evolutionary distances from (well-studied) eudicots or angiosperms. Overall, their messages are clear; this work will be an important warning for end users and encourage the developer of prediction algorithms to address this issue.

Thanks, very much appreciated.

1. Although the claim in the title is likely correct, they should claim only what they showed in this study.

Fair point, we have edited the title, which now reads:
'Reliability of plastid and mitochondrial localisation prediction declines rapidly with the evolutionary distance to the training set increasing'

2. The authors seem to presume that all nuclear-encoded plastid/mitochondrial proteins carry N-terminal targeting sequences. However, this assumption has not been proven (if I understand correctly). If this assumption is incorrect, we cannot expect TargetP to be 100% accurate because it only predicts the existence/absence of N-terminal signals. This point should be noted clearly.

Thanks for mentioning this. Import independent of N-terminal targeting sequences is indeed more frequent than often assumed (and was part of our own previous research), and we now address this more clearly (lines 362-366).

3. More importantly, their main message, "Performance of localization prediction algorithms decreases rapidly with the evolutionary distance …," seems to imply the existence of a general/ideal predictor applicable to the sequences of any species. However, as the authors point out, the translocation complexes can vary between species. The efficiency of recognition of a typical signal in one species can be greatly different in another species. Therefore, the only reasonable solution seems to prepare specific predictor for each evolutionary group of species. Using machine learning techniques, such a remedy would be rather trivial (if there is enough data size).

While it is a reasonable first step to develop specific predictor for each evolutionary group of species – as sometimes the case and examples cited by us – recent advances in machine learning in combination with many new genomes now published, offer the chance for the identification of higher order patterns. Our work identifies some current pitfalls and discusses (now more so), how new algorithms might be improved by those working on them.

4. In this sense, the authors should show how different the N-terminal signals are between different evolutionary groups (in the case of mitochondria, animal sequences should also be compared) (like Fig.4c & d).

Differences between animal and plant mitochondrial sequences are known to an extent as we already cite and the same is true between algae and angiosperms. The latter is less appreciated in the literature and a high-resolution analysis of NTSs from algae to angiosperms would require a study of its own.

5. Although this does not matter for users, Wolf PSORT was released 12 years before TargetP v.2.0. Obviously, there was not much sample data for various species at that time. So, at least from the side of algorithm developers, the comparison ignoring this point does not make much sense.

The only reason why we put TargetP next to WolfPSORT is the frequency with which they are used/cited. We fully agree that for its time it was up to date (and a go-to and precious tool for many including us) and only more recently, the other programs are a generation ahead in terms of species and technique. We make this distinction explicit in the introduction now.

6. The current explanation of the Localizer algorithm should be improved. The first version of PSORT also could treat eukaryotic sequences (PMD 1478671).

Thanks for this clarification. Done.

Reviewer 2

We are thankful to R2 for the literature and perspectives offered. Addressing the comments has improved the text, especially with respect to the necessity of our analyses.

Evident across R2 comments, is the question of how one defines ‘diversity’ across major taxonomic branches of life. Several model systems (or species otherwise relevant to human activity) are often mainstream, yet the vast diversity next to those are usually neglected (e.g., the “plant kingdom” is often equated to *Arabidopsis*, crops and maybe some other dicots). To us, and researchers interested in the diversification of major lineages and early evolution, the diversity of a kingdom must touch upon all branches since its very origins (for the plant kingdom this means including all branches since the origin of the plastid or at minimum since the origin of land plants). This perspective differentiates our analyses from the existing ones; we elaborate this in the text and below in response to the comments.

Reviewer #2: The study aims to clarify the prediction error of plastid and mitochondrion proteomes across the plant kingdom with evolutionary distance from the training standard and speculate ways to improve prediction accuracy/precision. It is a well written manuscript. While this is an ongoing issue of high relevance the novelty of the study is limited and concrete steps for improvements are not given.

Overall, the claim that the proteomes predicted and experimental vary due to errors and differences in plant physiology across evolution are known. Secondly, the prediction algorithms are mostly trained on e.g. Arabidopsis and will work best on this species as there has been the most data to train. So this is not a novel insight.

We agree (see also the first paragraph of the cover letter of the original submission) that a certain unreliability of algorithms is known, albeit likely restricted in the broader sense to the community of protein targeting. These algorithms, however, are used by many more and it remains a very common request from peers (in peer review and at conferences) that one should use these algorithms no matter the species in question. A message that these

algorithms are unreliable outside of the training species for reasons we define, is needed and is best communicated in the published record.

Our analysis covers all species with archaeplastidal plastids of primary origin, where an evolutionary perspective also helps us quantify how unreliable a prediction is as a function of evolutionary distance from the training species. We also reflect on sources of errors in light of simultaneous presence of plastid-mitochondria and cross-organelle errors therein. We trust that our study is novel in its overall perspective, approach to the benchmarking, breadth of species, directness of the message regarding their unreliability, providing a timely reminder to an ambitious goal of generalised prediction algorithm and the improvement strategies (which we now make explicit with added text and an additional figure in the discussion).

Abstract is not clearly stating that the study is limited to plastid and mitochondria and which 4 species were used to assess the predictors. The authors make claims using only Arabidopsis, Chlamydomonas, Zea mays, P patens to choose the best predictor. There are a lot more experimental data out there so the authors could do a more substantial analysis across several individuals computationally and then also using experimental data sets.

The title is now more explicit and the abstract clear on the range of species, along with the rationale for selecting them. It is a common practice in evolutionary biology to use a subset of key species from as many diverse clades as possible, instead of taking many (or all available) from the same clade. The diversity, not the sheer number of species, matters. It is already evident from the evolutionary corelation (Fig. 3) that the reliability in angiosperms is high (e.g. a precision of 0.7-0.8 for TargetP predicting plastid proteins) and adding more angiosperm organelle proteomes will not add much new information; more bryophyte and algal organelle proteomes, however, will.

However, the authors need to compare their assessment of 3 predictors with other assessments. For example, the comparison of >10 predictors against multiple experimental data sets and classifiers in Arabidopsis (e.g. SUBAcon and/or the SUBA4 (Bioinformatics. 2014 Dec 1;30(23):3356-64. doi: 10.1093/bioinformatics/btu550. Epub 2014 Aug 22.). The latter had a full assessment of ~20 predictors including TargetP and WolfPSORT. This paper also found a TargetP to be one of the strongest predictors for plastid using a far more rigid methodology for comparing to experimental data alone.

Such analyses and a general notion regarding performance also influenced our choice of the three algorithms in the first place. This is now better phrased in the discussion (lines 286-288). SUBA is an important benchmark that we now discuss, too (lines 98-99, 301-303, 345-346). These benchmarks cover diverse algorithms, but, again, not clades. A goal of the current study is to cover a wider evolutionary diversity of species, at the whole genome scale, which limited the number of algorithms to consider.

The authors may also need to consider Predotar (Proteomics. 2004 Jun;4(6):1581-90. doi: 10.1002/pmic.200300776.) which is still a strong predictor for mitochondria using NTS and the training set may be more widespread perhaps.

Predotar only takes into account “higher plants” and tests on a “higher plant” i.e. *Arabidopsis*. We expect it to perform poorly outside of angiosperms, however, we could not test this as we could not access a command line version (suitable to the whole genome analysis) of Predotar. The link in the original publication is no longer functional, emails to the repository were not delivered, and we were also unable to send requests via the repository website. We remain open to this algorithm (or any other), should a command line version become accessible to the public.

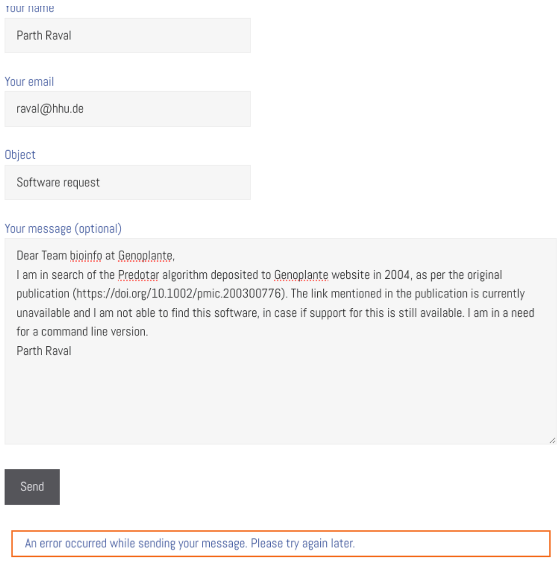
Your name

Parth Raval

Your email

raval@hhu.de

Object

Software request

Your message (optional)

Dear Team bioinfo at Genoplante,
I am in search of the Predotar algorithm deposited to Genoplante website in 2004, as per the original publication (https://doi.org/10.1002/pmic.200300776). The link mentioned in the publication is currently unavailable and I am not able to find this software, in case if support for this is still available. I am in a need for a command line version.
Parth Raval

Send

An error occurred while sending your message. Please try again later.

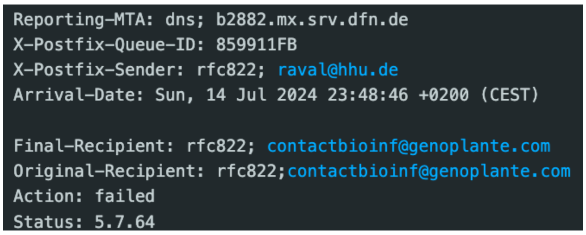
```
Reporting-MTA: dns; b2882.mx.srv.dfn.de
X-Postfix-Queue-ID: 859911FB
X-Postfix-Sender: rfc822; raval@hhu.de
Arrival-Date: Sun, 14 Jul 2024 23:48:46 +0200 (CEST)

Final-Recipient: rfc822; contactbioinf@genoplante.com
Original-Recipient: rfc822;contactbioinf@genoplante.com
Action: failed
Status: 5.7.64
```

Secondly, the cropPAL dataset mentioned by the authors has 12 species including experimental data and was used to describe the divergence of the subcellular proteomes to some degree across 6 monocots and 6 dicots from different branches of the plant kingdom (Plant J. 2020 Nov;104(3):812-827. doi: 10.1111/tpj.14961. Epub 2020 Sep 16). Can the authors discuss this and emphasis how their claim is different or more substantial?

Monocots and eudicots, while highly relevant to crop sciences, are all angiosperms. For an analysis confined to angiosperms, cropPAL dataset and benchmark is comprehensive – better explained now in our revision (lines 380-384) – and we also use it to probe dual targeting to plastid and mitochondria (lines 343-345). As to differentiate our analyses, the plant kingdom, especially when defined by a presence of chloroplast, is much more diverse. As we emphasize in the discussion, algae and bryophytes are of critical to global ecosystems and part of fundamental research more and more, too. Our goal is to expand the taxonomic and evolutionary scope of benchmarking, and thereby define the angles from which to improve, considering the known diversity of plants. To this end, cropPAL is certainly an endeavour with lessons to learn from, as also now discussed (lines 300-303).

Also, the experimental sets used to verify predictors need to be described in more detail. What were the QC parameters in this data set. The authors assume that these are correct and maybe complete. Experimental data sets are also error prone to contamination and missing proteins. If using MS data, this method tends to detect similar protein families across species, namely prevalent proteins and those that form detectable peptides. Hence all proteomes generated by MS tend to plateau towards the same group of but always miss the rest of proteins. In that light, the authors state a large number of false positives but to what degree is this correct? The experimental plastid/mitochondria datasets are much smaller than expected so a lot of the false positives may actually be correct? Another point not discussed by the authors is how experimental discrepancies are being dealt with?

Experimental datasets were not collected by us. QC parameters are hence as per the original studies or database criteria (generally at least one peptide per sample and as per the accepted Mascot norms). We do not assume these experimental data are complete or correct, we now mention this more explicitly (lines 299-303). Dealing with experimental

discrepancies (e.g. amongst different experimental techniques) requires projects such as SUBA, to be conducted across species.

Most literature expect plastid and mitochondrion proteomes to be around 10-15%. The authors mention 5%. The evidence for this needs to be described.

Point taken. 5% was a rather conservative, lower bound that we rephrased. Thanks.

The evolutionary approach is very interesting and more convincing and as mentioned earlier I would not readily assume that the differences seen in the prediction are errors. Can the authors clarify that the prediction error definition is? Is it prediction compared to experimental? If so, what about missing experimental proteins that are in the plastid/mitochondrion?

The prediction error is defined as a lack of overlap against experimental proteomes and we are now explicit on the possibility that the false positive could include experimentally missed out proteins (lines 299-300). We toned down the language likewise (lines 297-298).

Proteins are known to be conserved as well as in distinctly different organelles amongst different species. The authors show the training set for TargetP per species but what is the distribution of the training standard compared to the test data sets of 147 species? Is it comparable or is the training standard skewed differently to the test? This needs to be clearer in the text.

Taxonomic classification of 147 test species is communicated more clearly now in Fig. 3 and Fig. S5.

There are several individual predictors and ensemble predictors/classifiers that take evolution into account. These have not been considered or discussed (e.g. BaCelLo - Bioinformatics. 2006 Jul 15;22(14):e408-16. doi: 10.1093/bioinformatics/btl222.). How do more balanced predictors perform?

Thanks for the suggestion, we now complement a part of our analyses with BaCelLo (lines 324-327).

The results suggest also that apart from distance there are other relationships limited to e.g. clades etc. Which is interesting and may warrant more investigation as this has not been done systematically before. The authors suggest an algorithm with better adjustment to evolutionary distance but do not propose how this would work.

We now elaborate more explicitly on how to improve future algorithms in the discussion (lines 353-362, Fig. 6)

Choosing protein families is an interesting approach as such and it leads to the conundrum to include families with proteins only existent in the plastid or in the mitochondria versus families with proteins that have members sent to different locations. How are these considered? This may have introduced a lot of missed proteins that are not really missed.

To account for this, for each protein family (consisting of several proteins from many species), and even if only a single protein is organelle localised in a single species, we included all its members when we compare them against the predictions. This indeed overestimates the organelle proteins inferred based on protein family approach to some degree (e.g. the members localised elsewhere), but importantly the verification of the predicted proteins does

not miss these cases. We do not delve into ‘false negative’ hereby created (i.e. proteins inferred to be organelle localised based on protein family approach, but absent from experimental organelle proteomes).

Pan proteome and genome training would be good idea but how to is not described.

We now elaborate more explicitly on this (lines 267-272).

Dual targeted proteins: This has been looked at a few times (e.g. in the cropPAL data set and in Arabidopsis versus Zea mays or Oryza and other species to some degree) and should be discussed here. In general, most predictors struggle predicting dual targeted proteins even if training on the species they are predicting. This is a long-standing issue and yet has not been well addressed in this study! The authors need to discuss the literature (e.g. New Phytol. 2009;183(1):224-236. doi: 10.1111/j.1469-8137.2009.02832.x.).

Thanks for the suggestions. We now include dual targeting predicted vs. verified by cropPAL for six species out of twelve (the other six showed no experimentally verified dual targeted proteins and were excluded) and discuss SUBAcon (lines 343-346). We are aware of the ambiguous targeting predictor, but unfortunately could not find it online which made it impossible to include it in our analysis. We now benchmark and discuss its predictions from the original publication for *Arabidopsis* (lines 341-343).

Other comments:
Figures need stand-alone legends. Please review each and add missing explanations particularly the supplementary.

We have now added relevant details to the legends.

Reviewer 3

Reviewer #3: The paper titled "Performance of localization prediction algorithms decreases rapidly with the evolutionary distance to the training set increasing" presents a very interesting and systematic evaluation to plant organelle proteomes. The study took a big collection of known plant organelle proteome data as a basis to evaluate three major computational prediction tools. I think this is a good study. I also personally favor its conclusion. I have only two minor comments, as follows:
1 As this is computational biology, I think the authors should present the major mathematical formulations in the main text, not only in supps.
2 I think the code and datasets for reproducing the study should also be deposited publicly. Zenodo and Github are recommended.

Thank you for the encouraging comments. While it is a computational biology submission, since it evaluates available algorithm, there are no major mathematical formulations to be included in the main text. The codes and datasets are all available on Zenodo.
