# Supplementary material for: Reliability of plastid and mitochondrial localisation prediction declines rapidly with the evolutionary distance to the training set increasing
Source: PLoS Comput Biol. 2024 Nov 11;20(11):e1012575. doi: 10.1371/journal.pcbi.1012575 (PMC11581415; doi:10.1371/journal.pcbi.1012575)
Supplement: Attachment — Submitted filename: Response to Reviewers_Rev2.docx [file pcbi.1012575.s024.docx]

**Reviewer 1**

Reviewer #1: Since I confirmed that the authors' responses to my points are reasonable, I now recommend its acceptance as is.

Thank you for your valuable input. We have now also updated our Zenedo submission including additional files, codes and an instruction file (also in line with R3’s comments).

**Reviewer 2**

Reviewer #2: The authors have put substantial work into improving the manuscript. It is now much clearer that this study focusses on plastid and mitochondria across cornerstones of the plant kingdom. I agree that many prediction algorithms are not accessible 5 or more years after publication, highlighting a serious issue in our system. The authors also adjusted and increased their conclusions substantially to reflect the data limitations and findings.

Thanks for recommending the relevant literature and useful additional analyses, all of which were incorporated and significantly improved our manuscript. A sincere Thank you.

**Reviewer 3**

Reviewer #3: I do not think the authors face my questions and concerns with a consolidated scientific response. I believe none of my concerns is addressed. In particular, no reproducible instruction is provided in zenodo. The codes and XLS files are thus useless. Therefore, I am sorry that I suggest to reject this paper because the background information (data/codes/instructions for reproducing) is not fully published.

We apologise that our Zenedo submission was found to be insufficient. It is a difficult line between drowning the readers through too much information and providing too little data. We therefore provided the source data for all figures and the key codes in the Zenedo submission.

We agree that background data is crucial and needs to be available, especially amidst the reproducibility crisis. We have created a new Zenedo upload which also includes a step-by-step instruction file (along with references to the codes and the data tables). The scripts are now referred to in the methodology section, too.

For reasons of transparency, we copy your comments from the first revision and readdress them here:

The paper titled "Performance of localization prediction algorithms decreases rapidly with the evolutionary distance to the training set increasing" presents a very interesting and systematic evaluation to plant organelle proteomes. The study took a big collection of known plant organelle proteome data as a basis to evaluate three major computational prediction tools. I think this is a good study. I also personally favor its conclusion. I have only two minor comments, as follows:

1 As this is computational biology, I think the authors should present the major mathematical formulations in the main text, not only in supps.

As such there are no new mathematical formulations/theory/algorithm that we develop, as our work benchmarks pre-existing algorithms. This benchmarking involved traditional mathematical formulations that we now included in the methods.

2 I think the code and datasets for reproducing the study should also be deposited publicly. Zenodo and Github are recommended.

A figure-by-figure instruction file is now added on Zenedo for the reproducibility (https://zenodo.org/records/13924211).
